# DNA-interacting properties of two analogous square-planar *cis*-chlorido complexes: copper versus palladium

**DOI:** 10.1007/s00775-021-01888-2

**Published:** 2021-08-28

**Authors:** Marcos V. Palmeira-Mello, Ana B. Caballero, Aida Lopez-Espinar, Guilherme P. Guedes, Amparo Caubet, Alessandra M. Teles de Souza, Mauricio Lanznaster, Patrick Gamez

**Affiliations:** 1 Instituto de Química, Universidade Federal Fluminense, Outeiro S. João Batista S/N, Niterói, RJ 24020−141 Brazil; 2 nanoBIC, Departament de Química Inorgànica i Orgànica, Secció Química Inorgànica, Universitat de Barcelona, Martí i Franquès 1−11, 08028 Barcelona, Spain; 3 Laboratório de Modelagem Molecular and QSAR (ModMolQSAR), Faculdade de Farmácia, Universidade Federal do Rio de Janeiro, Rio de Janeiro, RJ Brazil; 4 Institute of Nanoscience and Nanotechnology (IN2UB), Universitat de Barcelona, 08028 Barcelona, Spain; 5 Catalan Institution for Research and Advanced Studies (ICREA), Passeig Lluís Companys 23, 08010 Barcelona, Spain

**Keywords:** Copper(||), Palladium(||), Cisplatin, DNA interaction, Cleavage, Molecular docking

## Abstract

Two square-planar coordination compounds, namely [Cu(CPYA)Cl_2_] (**1**) and [Pd(CPYA)Cl_2_] (**2**), were prepared from the ligand 4-chloro-*N*-(pyridin-2-ylmethyl)aniline (CPYA) and two chloride salts, and were fully characterized, including by X-ray diffraction. Spectroscopic, electrophoretic and AFM studies revealed that the two isostructural compounds were interacting differently with DNA. In both cases, the initial interaction involves electrostatic contacts of the CPYA ligand in the minor groove (as suggested by molecular docking), but subsequent strong binding occurs with the palladium(II) complex **2**, whereas the binding with the copper complex **1** is weaker and concentration dependent. The strong binding of **2** eventually leads to the cleavage of the double strand and the redox activity of **1** allows to oxidatively cleave the biomolecule.

**Graphic abstract:**

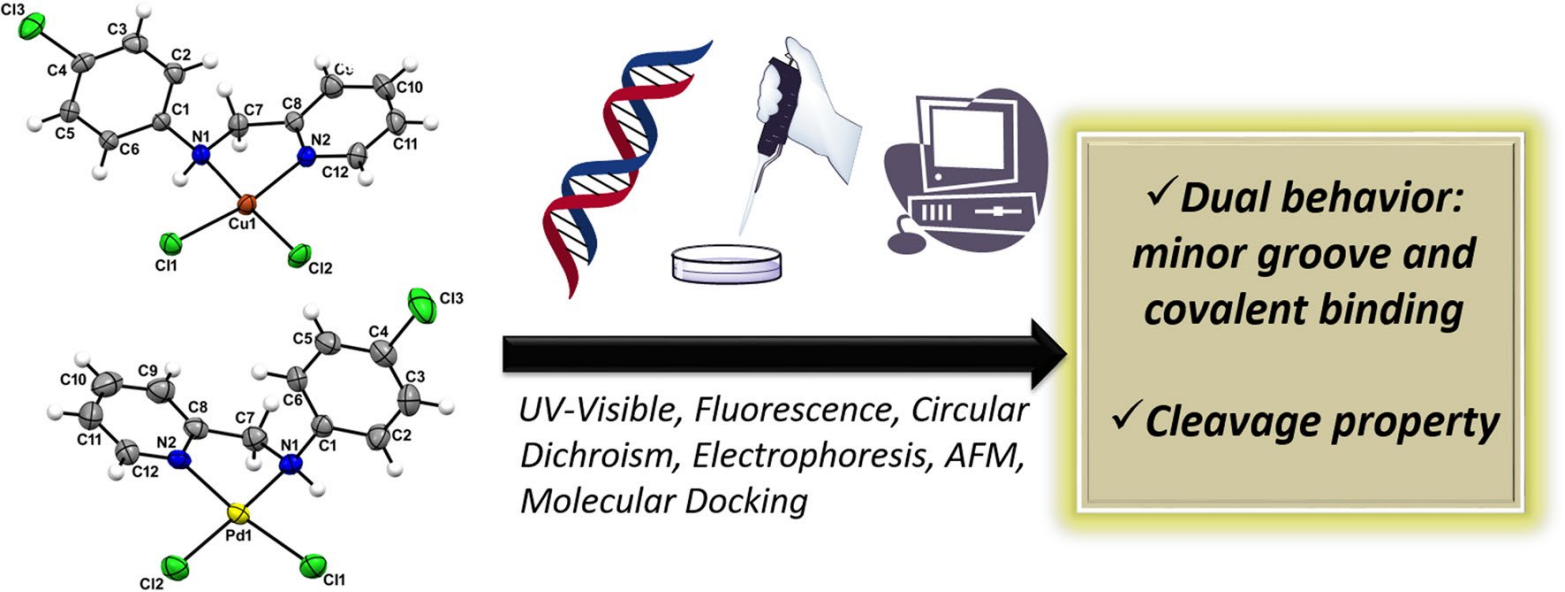

**Supplementary Information:**

The online version contains supplementary material available at 10.1007/s00775-021-01888-2.

## Introduction

Since the discovery of *cis-*[PtCl_2_(NH_3_)_2_] (cisplatin), DNA has been a key target in the development of metal-based antitumoral drugs [1, 2]. Platinum(II) complexes, such as cisplatin, are employed in the treatment of several types of cancer, with a mechanism of action that involves binding to purine DNA base pairs, mainly at position N7 of guanine [3]. Despite their anticancer efficacy [4, 5], platinum-containing compounds lack specificity and are frequently accompanied by drug resistance and severe side effects, such as nephrotoxicity. Therefore, several coordination compounds based on other metal ions have been investigated as an alternative for platinum in the development of new antitumor agents, with potentially distinct mechanisms of action [6–11].

Copper is an essential biological trace element, which plays a vital role in biological systems, for example, in dioxygen transport and electron-transfer processes. Its DNA-cleaving properties via hydrolytic or oxidative pathways make copper-based complexes attractive candidates in the development of new anticancer agents [12, 13]. While the former pathway involves phosphodiester hydrolysis induced by the metal-based compound, the second one is associated with singlet dioxygen, reactive oxygen species (ROS) or electron transfer, which may cause modifications on nucleobases or the deoxyribose skeleton, preventing the progression of tumor cells [12].

Unlike copper, there is no evidence regarding any biological functions of palladium [14]. DNA-binding studies of palladium-based complexes have been investigated [15–19] due to their structural similarities with Pt(II) compounds. Indeed, a square-planar geometry is favored for both metal ions; however, the ligand-exchange kinetics is faster for palladium compounds, generating more reactive compounds that may interact with pharmacological targets in distinct ways [20–22].

In this context, a number of copper(II) and palladium(II) compounds have been investigated as potential DNA binders and/or cleavers [17, 18, 23–25]. In a previous study, we investigated the DNA-interacting and cytotoxic properties of a dinuclear [Cu(*L*)Cl_2_]_2_ complex, where *L* is a Schiff base ligand, namely (*E*)-phenyl-*N*-((pyridin-2-yl)methylene)methanamine [26]. DNA-binding studies revealed that mononuclear species, formed in solution through breakage of the chloride bridge, were acting as DNA cleaver. This compound also presented a higher cytotoxicity than its platinum(II) analog [Pt(*L*)Cl_2_].

**Fig. 1:**
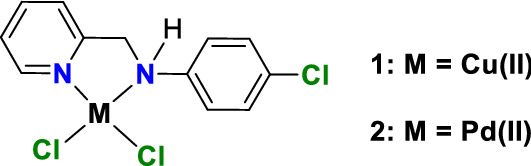
Structures of the complexes [Cu(CPYA)Cl_2_] (**1**) and [Pd(CPYA)Cl_2_] (**2**)

Bidentate N-donor ligands are commonly used in coordination chemistry [27, 28]. In the present study, two square-planar complexes, viz. [Cu(CPYA)Cl_2_] (**1**) and [Pd(CPYA)Cl_2_] (**2**) (CPYA = *N,N*-4-chloro-*N*-(pyridin-2-ylmethyl)aniline), were synthesized and fully characterized (Fig. 1). The DNA-interacting properties of **1** and **2** were thoroughly examined with various techniques, including molecular docking studies.

## Experimental

### Materials and instrumentation

All starting materials were purchased from commercial sources and used without further purification. The ligand 4-chloro-*N*-(pyridin-2-ylmethyl)aniline (CPYA) and the complex precursor Na_2_[PdCl_4_] were synthesized and characterized following reported procedures [29, 30]. Infrared spectra were measured from 4000 to 600 cm^–1^ on a Varian 660 FTIR equipped with Pike Miracle diamond/ZnSe ATR. ^1^H NMR spectra were recorded in DMSO-d_6_ at room temperature, using a Varian Unity-Plus 500 MHz spectrometer. Chemical shifts (*δ*) are given in ppm. CHN microanalyses were performed at the Universidade de São Paulo, Brazil, using a Perkin-Elmer CHN 2400 micro analyzer and with a Fisons EA 1108 analyzer at the Universitat de Barcelona, Spain. ESI–MS data were collected with a Perkin-Elmer SQ-300 mass spectrometer by direct infusion using MeOH (MS grade) as solvent.

For the DNA-interaction studies, sodium cacodylate, NaCl, Tris–borate–EDTA (TBE), ethidium bromide (EB), Hoechst 33258, pBR322 plasmid DNA (0.5 µg µL^−1^), calf thymus DNA (ct-DNA) and SYBR™ Safe DNA Gel Stain (Invitrogen) were acquired from commercial sources and used without further purification. Ultrapure Milli-Q^®^ water was used to prepare the solutions.

### Synthesis

A methanolic solution (10 mL) of CPYA (437 mg, 2.0 mmol) was added to a solution of CuCl_2_·6H_2_O (340 mg, 2.0 mmol) or Na_2_[PdCl_4_] (588 mg, 2.0 mmol) in 10 mL of methanol; the resulting mixture was stirred for 30 min at 40 ºC. The formed precipitate was filtered out, washed with diethyl ether and dried under reduced pressure.

[Cu(CPYA)Cl_2_] (**1**): green powder, yield: 44%. IR (ATR, cm^−1^): 3189 m (N−H), 2923 w (C−H), 1608 s, 1490 s (C=C, C=N). Anal. calc. for C_12_H_11_Cl_3_N_2_Cu: C, 40.82; H, 3.14; N, 7.93%. Found: C, 40.65; H, 3.03; N, 7.84%. ESI–MS (MeOH): *m/z* (positive mode) = 316 ([Cu(CPYA)(H_2_O)(OH)]^+^).

[Pd(CPYA)Cl_2_] (**2**): yellow powder, yield: 60%. IR (ATR, cm^−1^): 3189 m (N−H), 2934 w (C−H), 1608 s, 1490 s (C=C, C=N). ^1^H NMR (500 MHz, dmso-d_6_) δ, ppm: 8.91 (d, 1H), 8.75 (d, 1H), 8.17 (t, 1H), 7.76 (d, 1H), 7.60 (t, 1H), 7.39 (d, 2H), 7.15 (d, 2H), 4.92 (dd, 1H), 4.47 (dd, 1H). Anal. calc. for C_12_H_11_Cl_3_N_2_Pd: C, 36.40; H, 2.80; N, 7.07%. Found: C, 36.57; H, 2.73; N, 6.87%. ESI–MS (MeOH): *m/z* (positive mode) = 395 ([Pd(CPYA)Cl_2_] + H]^+^).

### Single-crystal X-ray diffraction

Single crystals were obtained from recrystallization in methanol (**1**) or acetonitrile (**2**). X-ray diffraction data were collected on a Bruker D8 Venture diffractometer equipped with CMOS detector and using MoKα radiation (*λ* = 0.71073 Å) at room temperature. Data collection, cell refinement and data reduction were performed with Bruker Instrument Service vV6.2.6, APEX3 and SAINT, respectively [31, 32]. The absorption correction using equivalent reflections was done with the SADABS program [33]. The structure solutions and full-matrix least-squares refinements on *F*^2^ were performed with SHELXS [34] and SHELXL programs [35], respectively, implemented in OLEX2 [36]. All atoms except hydrogens were refined anisotropically and hydrogen atoms were treated using a constrained refinement. Structure drawings were generated using mercury [37]. Supplementary crystallographic data for this paper have been deposited with the CCDC: 2077060 (**1**) and 2077061 (**2**); these data can be obtained free of charge via http://www.ccdc.cam.ac.uk, or by contacting the Cambridge Crystallographic Data Centre, 12, Union Road, Cambridge CB2 IEZ, UK; Tel.: + 44 1223 336408; Fax: + 44 1223 336003.

### DNA-binding studies

For the DNA-binding studies, the concentration of calf thymus DNA (ct-DNA, Sigma-Aldrich) was determined spectrophotometrically at 260 nm using the nucleobase molar absorptivity of 6600 M^−1^ cm^−1^. A cacodylate–NaCl buffer solution containing 1 mM cacodylate and 20 mM NaCl at pH 7.3 was used.

#### UV–Vis spectroscopy

The electronic spectra were recorded in cacodylate–NaCl buffer solutions with a Varian Cary 100 spectrophotometer at room temperature, using a 1 cm path length cuvette. The complexes were dissolved in DMSO and the final solutions contained 1% DMSO. The samples were incubated in cacodylate–NaCl buffer at 37 °C for 1 h (copper complex **1**) or 24 h (palladium complex **2**). To study the binding to DNA, a 25 µM solution of metal complex was incubated for the same time periods as for the stability tests with increasing concentrations of ct-DNA (0–50 μM_bp_, i.e., referred to base pairs) while keeping constant the concentration of the metal complex. The intrinsic binding constant, *K*_b_, was obtained using Eq. (1).

1 $$ \frac{{\left[ {{\text{DNA}}} \right]}}{{\left( {\varepsilon_{a} - \varepsilon_{f} } \right)}} = \frac{{\left[ {{\text{DNA}}} \right]}}{{\left( {\varepsilon_{0} - \varepsilon_{f} } \right)}} + \frac{1}{{K_{b} \left( {\varepsilon_{0} - \varepsilon_{f} } \right)}}, $$

where [DNA] is the concentration of ct-DNA in base pairs, *ε*_*a*_ is the extinction coefficient observed at the given DNA concentration, *ε*_*f*_ is the extinction coefficient of the free complex in solution, and ε_0_ is the extinction coefficient of the compound when fully bound to DNA.

#### Fluorescence spectroscopy

Emission intensity measurements were carried out using a HORIBA Jobin–Yvon iHR320 spectrofluorometer at room temperature. The solution of ct-DNA (15 μM_bp_, i.e., referred to base pairs) was pre-incubated with the intercalating agent ethidium bromide (EB, 75 μM), or with the minor groove binder Hoechst 33258 (2 μM) in cacodylate–NaCl buffer for 30 min at 37 °C, to allow its full interaction with the dye. Increasing amounts of **1** and **2** (0–50 μM) were added to this mixture and the final samples were incubated for 1 h (copper complex **1**) or 24 h (palladium complex **2**). After incubation, the emission spectra were registered upon excitation at 514 nm for the measurements with EB, and 350 nm for Hoechst 33258. The quenching data were analyzed applying the Stern–Volmer equation (Eq. 2).

2 $$ \frac{{I_{0} }}{I} = 1 + K_{SV} \left[ Q \right] ,$$

where *I*_0_ and *I* represent the fluorescence intensities of the DNA–dye complex in the absence and presence of quencher, respectively. *K*_*sv*_ is the linear Stern–Volmer quenching constant and [*Q*] is the concentration of the added metal complex.

#### Circular dichroism (CD) spectroscopy

The CD titrations were carried out with a JASCO J-815 spectropolarimeter at room temperature. Solutions of ct-DNA (100 μM) in cacodylate–NaCl buffer with different complex-to-DNA concentration ratios (0.0, 0.2, 0.6 and 1.0) were incubated at 37 °C for 1 h (copper complex **1**) or 24 h (palladium complex **2**), and the spectra of these solutions were recorded from 230 to 320 nm using a quartz cuvette with an optical path length of 0.5 cm and a scanning rate of 200 nm min^–1^.

#### Agarose gel electrophoresis

The nuclease activities of **1** and **2** were subsequently investigated by agarose gel electrophoresis using pBR322 plasmid DNA. Stock solutions of **1** and **2** (10 mM) were prepared in cacodylate–NaCl buffer. In addition, the DNA-cleaving compound [Cu(phen)_2_(H_2_O)](NO_3_)_2_—also known as Sigman’s reagent—and the covalent binder to DNA cisplatin were used as reference compounds for the assays. The former was synthesized as previously reported [38]. The plasmid pBR322 was treated with different concentrations of compounds (5–100 µM) and the samples were incubated at 37 °C for 1 h (copper complex **1**) or 24 h (palladium complex **2**). Then, the samples were loaded onto the gel with 4 μL loading buffer (30% glycerol, 5 mM xylene cyanol) and electrophoresed in TBE 1X at 100 V for 1 h. For **1**, the analysis was also performed in the presence of ascorbic acid (AA, 100 µM) as a reducing agent. The electrophoresis was run in a Bio-Rad horizontal tank at 6.25 V cm^−1^. Next, the gel was stained with SYBR™ Safe overnight and the images were acquired using a Gel Doc EZ Imager instrument (Bio-Rad). The percentage of DNA–cleaving activity was calculated according to Eq. (3).

3 $$ \% {\text{DNA}} - {\text{cleaving activity}} = \left( {\frac{{\left[ {{\text{Form}} {\text{II}}} \right]}}{{\left[ {\text{Form I}} \right] + \left[ {\text{Form II}} \right]}}} \right) \times 100. $$

#### Atomic force microscopy (AFM)

pBR322 plasmid DNA aliquots (5 and 25 µg mL^–1^) in HEPES–MgCl_2_ buffer (40 mM HEPES and 10 mM MgCl_2_) were incubated at 60 ºC for 10 min. Immediately after, the metal compounds were added and incubated with open DNA for 1 h (copper complex) and 24 h (palladium complex) at 37 °C. The AFM samples were prepared by casting a 16 μL drop of test solution onto freshly cleaved muscovite green mica disks as the support. The drop was allowed to stand for 20 s to favor the adsorbate–substrate interaction. Each DNA-laden disk was rinsed gently with Milli-Q water and was blown dry with clean compressed argon gas directed to the disk surface. The samples were prepared just prior to AFM imaging. Images were recorded with a Bruker AFM Multimode 8 with nanoscope V electronics using an SNL tip and ScanAsyst mode (1 Hz).

### Molecular docking

Molecular docking studies were performed using the AutoDock 4.2 program on a Windows-based PC [39]. The crystal structure of B-DNA dodecamer was obtained from the Protein Data Bank (http://www.rcsb.org/pdb/) under code 1BNA and 1.90 Å resolution. The three-dimensional structures of [Cu(CPYA)Cl_2_] (**1**) and [Pd(CPYA)Cl_2_] (**2**) were obtained from cif files of their X-ray crystal structures and converted into pdb format using Mercury (http://www.ccdc.cam.ac.uk/) [37]. The three-dimensional structures of aquo species [Cu(CPYA)(H_2_O)_2_]^2+^ (**1a**) and [Pd(CPYA)(H_2_O)_2_]^2+^ (**2a**) were obtained from **1** and **2** using the SPARTAN’10 Program (Wavefunction Inc., Irvine, CA, USA). The docking files were prepared using AutoDock Tools. The water molecules were removed, and DNA was treated by adding all hydrogens, then nonpolar hydrogen atoms were merged and Gasteiger charges were assigned by default. The metal parameters were added on pdbqt file of these compounds. The grid box was centered in the A–T–T–C region of the double helix and the parameters were set with 0.375 Å spacing and 40 × 40 × 40 points using AutoGrid program. Lamarckian Genetic Algorithm (LGA) was employed and a total of 100 runs were performed using the default parameters such as the initial population (150), number of energy assessments (2500000), mutation rate (0.02), crossover rate (0.8) and elitism (1). The results were analyzed using the PyMOL software package (The PyMOL Molecular Graphics System, Version 1.3, Schrödinger, LLC, San Francisco, CA, USA).

## Results and discussion

### Synthesis and characterization

Complexes [Cu(CPYA)Cl_2_] (**1**) and [Pd(CPYA)Cl_2_] (**2**) were synthesized by reactions between CPYA and CuCl_2_ or Na_2_[PdCl_4_] in methanol. The IR spectra of these complexes present a characteristic band at 3189 cm^−1^ assigned to the amine group (Figs. S1 and S2). This band is shifted upon metal coordination compared with that for the free CPYA ligand at 3365 cm^−1^ [29]. The ^1^H NMR spectrum of complex **2** (Fig. S3) in DMSO-d_6_ confirms its structure in solution. The electrospray ionization mass spectrometry (ESI–MS) spectrum of **1** obtained in methanol reveals ligand exchange of the labile chlorides. MS measurements indeed suggest the presence of several species with distinct coordination numbers, showing peaks that can be ascribed to the molecular ions [Cu(CPYA)(H_2_O)(OH)]^+^ and [Cu(CPYA)(H_2_O)_2_(OH)]^+^ at *m/z* 316 and 334, respectively. For complex **2**, a peak corresponding to {[Pd(CPYA)Cl_2_] + H}^+^ was observed at *m/z* 395 (Figs. S4 and S5). The electronic spectrum of **1** recorded in DMSO presents two bands, at 300 nm (*ε* = 6612 M^−1^ cm^−1^) and 394 nm (*ε* = 959 M^−1^ cm^−1^), which can be assigned to π − π* and ligand-to-metal charge-transfer (LMCT) transitions, respectively (Fig. S6). An additional band at 800 nm (ε = 132 M^−1^ cm^−1^) is ascribed to ligand-field (d–d) transitions. The UV–Vis spectrum of complex **2** displays bands at 300 nm (ε = 7155 M^−1^ cm^−1^) and 390 nm (*ε* = 910 M^−1^ cm^−1^), which correspond to intraligand and metal-centered transitions, respectively. In addition, a shoulder is observed at 324 nm (*ε* = 4950 M^−1^ cm^−1^) (Fig. S7). Similar bands were observed for other related copper(II) and palladium(II) complexes [20, 40–45].

### X-ray crystallography

**Fig. 2:**
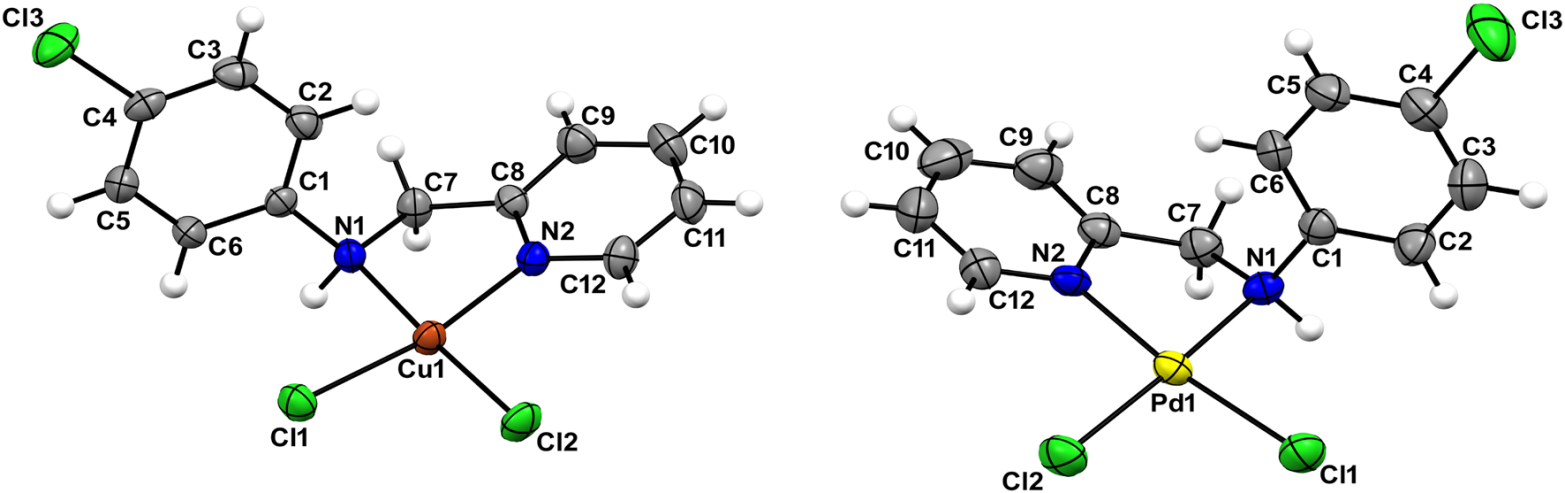
Thermal ellipsoids of [Cu(CPYA)Cl_2_] (**1**) (left) and [Pd(CPYA)Cl_2_] (**2**) (right). Atomic displacement parameters are drawn at the 50% probability level and hydrogen atoms are represented as spheres

Single crystals suitable for X-ray diffraction were obtained by slow evaporation of the solvent. Compounds **1** and **2** crystallized in the monoclinic *P*2_1_/c space group; representations of the crystal structures of **1** and **2** are depicted in Fig. 2. A summary of the crystal data, collection and refinement is gathered in Table S1, while selected bond lengths and angles are listed in Table S2. In both compounds, the metal ion (copper or palladium) lies on a slightly distorted square-planar environment, formed by one CPYA ligand coordinated in a bidentate fashion through the pyridine and amine nitrogen atoms, and to two chloride anions. The calculated Yang *τ*_4_ parameter for **1** and **2** is 0.11 and 0.08, respectively. The *τ*_4_ geometry index ranges from 0 for a perfect square-planar geometry to 1 for a purely tetrahedral geometry [46]. Similar structures were reported for copper and palladium complexes containing CPYA analogs with different substituents on the aromatic ring [27, 28].

The M-N1, M-N2 and M-Cl (average) bond lengths are of 2.087(2), 1.999(2) and 2.2585(7) Å for **1**, and of 2.081(2), 2.040(5) and 2.3060(17) Å for **2**. These bond lengths are in the typical range found for similar copper(II) and palladium(II)-based complexes [20, 41, 47]. The angle between the plane containing the atoms from the 4-chlorophenyl and the pyridine rings is 79.5º and 87.0º for **1** and **2**, respectively. Thus, the solid-state orientation of the 4-chlorophenyl ring with respect to the pyridine one is more orthogonal in **2**. A similar trend was observed for the angles between the plane formed by metal ion and the donor atoms, and the 4-chlorophenyl ring (72.6º for **1** and 72.0º for **2**).

Since both compounds crystallized in a centrosymmetric space group, both enantiomers can be seen in the crystal packing due to the inversion symmetry operation. The shortest metal distances are 4.0203(5) Å for **1** (Cu1^…^Cu1^i^, *i* = *x*, 3/2 − *y*, ½ + *z*) and 3.461(1) Å for **2** (Pd1^…^Pd1^i^, *i* = 1 − *x*, 1 − *y*, 1 − *z*). Hydrogen bonds (N–H^…^Cl and C_sp2_-H^…^Cl) involving the chlorides and the CPYA amine moiety contributed to stabilize the crystal packing of **1** and **2**. Further, an additional C_sp2_-H^…^N short contact among the [Pd(CPYA)Cl_2_] units and acetonitrile lattice solvent molecules is seen in **2**.

### DNA binding

Metal complexes can bind to DNA through several ways. Non-covalent interactions can occur via intercalation between two adjacent base pairs, via groove binding, or through electrostatic interactions with the sugar–phosphate backbone. Additionally, covalent binding can also occur with nucleophiles, and generally involves nitrogen or oxygen atoms of nucleobases [22].

Various means can be employed to investigate the interactions of a compound with DNA and elucidate the possible pathways that lead to its potential damage [48, 49]. In this work, DNA-interaction modes of **1** and **2** were investigated by UV–visible, fluorescence and circular dichroism spectroscopies.

#### Stability in solution

The stability of [Cu(CPYA)Cl_2_] (**1**) and [Pd(CPYA)Cl_2_] (**2**) in solution was examined by UV–visible spectroscopy. UV–Vis spectra were recorded during 1 h (complex **1**) or 24 h (complex **2**) in cacodylate − NaCl buffer containing 1% DMSO. As depicted in Figs. S8 and S9, some changes were observed for both complexes. ESI–MS data for **1** indicated the exchange of the chloride ligands with water and hydroxide. The observed changes in the UV–Vis spectra may result from such exchanges and may suggests that the species [Cu(CPYA)(H_2_O)(OH)]^+^ (formed from [Cu(CPYA)Cl_2_] in water) is that interacting with DNA. For **2**, the UV–Vis spectra show a decrease in the intensity of the band centered at ~ 400 nm, which can also be explained by the exchange of chloride ligands with solvent molecules (as observed for **1**). Time-resolved ^1^H NMR studies of **2** in DMSO/D_2_O solutions indicate that the chloride-exchanged palladium(II) species are stable for up to 24 h (Fig. S10). It can be concluded that the species interacting with DNA are most likely of the type [M(CPYA)(H_2_O)_2_]^2+^ or [M(CPYA)(H_2_O)(OH)]^+^.

#### UV–Vis spectroscopy

**Fig. 3:**
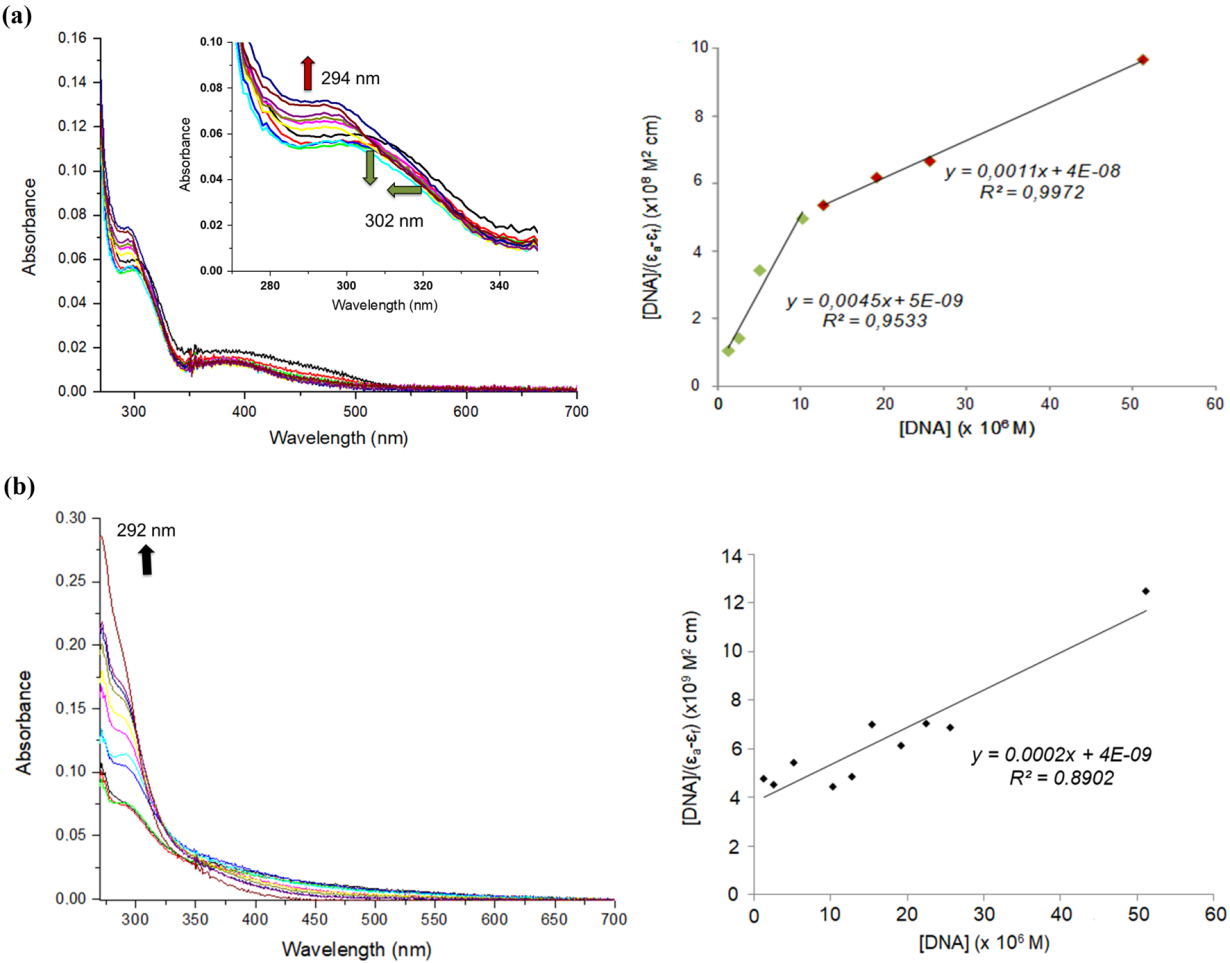
UV–Vis spectra of 25 μM solutions of **a** [Cu(CPYA)Cl_2_] (**1**) and **b** [Pd(CPYA)Cl_2_] (**2**) in the absence and presence of increasing amounts of ct-DNA (0 − 51.2 μM). The arrows show the intensity changes at the indicated wavelengths upon increase of [ct-DNA]. Measurements carried out in cacodylate–NaCl buffer after incubation at 37 °C for 1 h (**1**) and 24 h (**2**)

The electronic spectra of 25 μM solutions of **1** and **2** were recorded, respectively, after 1 h and 24 h of incubation with increasing concentrations of ct-DNA (Fig. 3). The spectral changes observed for **1** are consistent with two different binding modes. At higher complex-to-DNA ratios (down to 2.5), the π − π* charge-transfer band of the CPYA ligand (*λ* = 302 nm) shifts toward longer wavelengths along with a slight increase of the absorbance. Such effect may be attributed to non-covalent interactions between **1** and the biomolecule (e.g., via groove binding). These interactions probably involve the aromatic moieties of the CPYA ligand, as reflected by the bathochromic shift of the main band (Fig. 3a, inset). At complex:DNA ratios below 2, a marked blue shift of the band occurs, which is followed by a gradual and stronger hyperchromic effect with no further shift of the band. This feature may characterize an initial rearrangement of the complex–DNA interaction affecting the ligand aromaticity, to allow the coordination to the biomolecule, most likely to the nucleobases. The binding constant (*K*_*b*_) of **1** was estimated using Eq. (1). However, the data could only be fitted within two different ranges of complex:DNA ratios. One *K*_*b*_ value was obtained for the complex:DNA ratios corresponding to [DNA] = 12.8–51.2 µM, and another one for [DNA] = 0–10.2 µM. Hence, *K*_*b*_ values of 9.0 × 10^5^ M^−1^ (coordination binding) and 2.8 × 10^4^ M^−1^ (supramolecular binding) were obtained. For complex **2**, an increase in absorbance of the CPYA band at 292 nm is observed after addition of increasing amounts of DNA, without shift (Fig. 3b). Such substantial hyperchromic effect may result from a covalent bonding of the Pd(II) complex to nucleobases [50]. The corresponding binding constant is 5.0 × 10^4^ M^−1^. The *K*_*b*_ constants are comparable to those found for similar copper(II) and palladium(II)-based complexes [24, 27].

#### Dye-displacement fluorescence assays

**Fig. 4:**
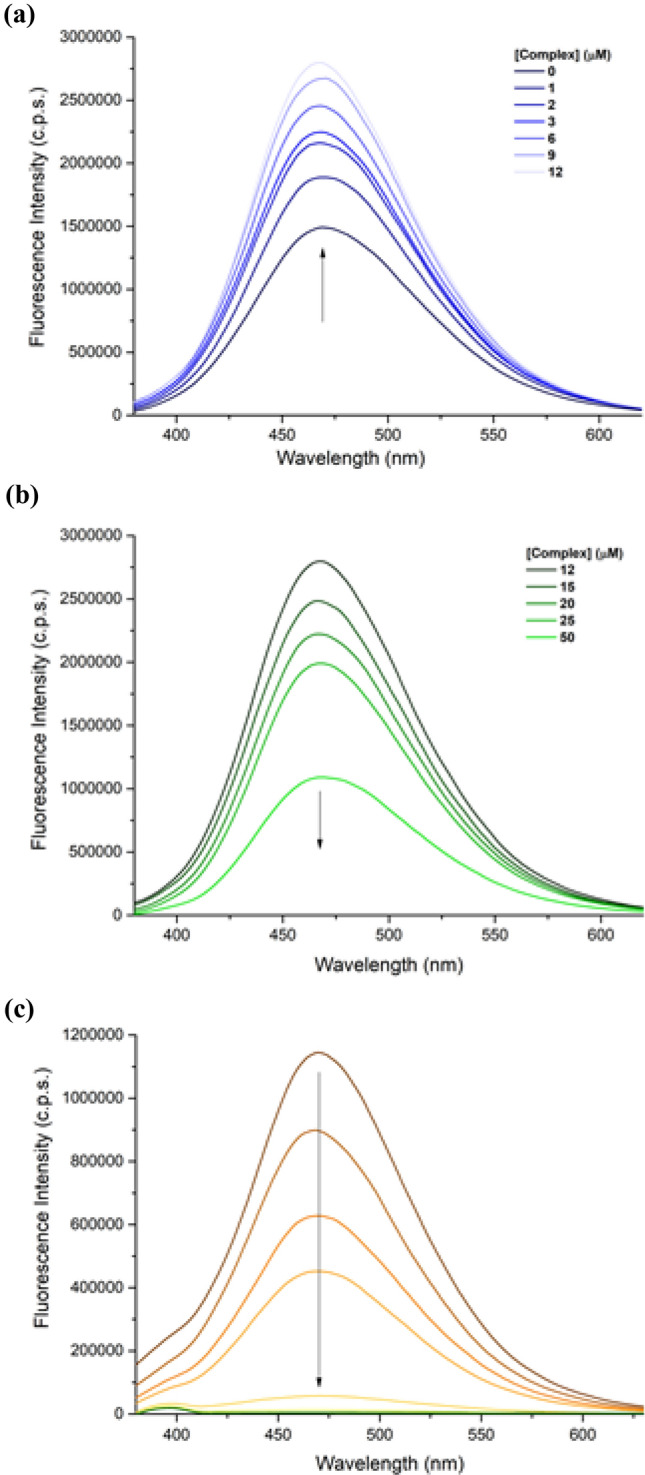
Fluorescence emission spectra of the DNA–Hoechst complex in the absence and presence of [Cu(CPYA)Cl_2_] **a** [**1**]  = 0–12 µM, **b** [**1**]  = 12–50 µM and [Pd(CPYA)Cl_2_], **c** [**2**]  = 0–50 µM; [Hoechst 33258] = 2 µM, [DNA] = 15 µM_bp_, *λ*_ex_ = 350 nm

To investigate further the interactions between DNA and complexes **1** and **2**, competitive binding studies with ethidium bromide (EB) were performed. EB is a DNA-intercalating agent that emits strongly at 610 nm upon excitation at 541 nm when bound to DNA. The binding displacement of this dye by a compound causes fluorescence quenching, which may be indicative of an intercalative behavior. In contrast to most reported copper(II) complexes [51–53], an increase of the emission was observed for **1** when increasing the concentration of complex; this may be explained by a higher hydrophobicity on the surroundings of EB, possibly as a consequence of compound-triggered conformational changes in the DNA structure. These observations exclude an intercalating behavior of **1** in the tested range of concentrations. No quenching of EB emission was observed in the case of **2**, indicating that this complex is not intercalating (Figs. S11 and S12). Since no displacement of the EB dye was observed for both complexes, competitive binding studies with the groove-binding dye Hoechst 33258 were subsequently performed (Fig. 4). Hoechst 33258 is a minor groove binder that fluoresces at 458 nm when bound to DNA upon excitation at 350 nm; its displacement by another compound results in fluorescence quenching. As for EB, an increase of the fluorescence intensity of Hoechst was observed for **1** at low complex:DNA ratios, most likely as a consequence of an increase of the hydrophobicity around the dye (Fig. 4a). However, a quenching was observed at higher complex:DNA ratios (above 1), indicating that **1** can eventually displace Hoechst 33258 from the biomolecule (Fig. 4b). In contrast, the emission intensity of the dye drastically decreased from the first addition of **2** (1 µM), therefore suggesting that the palladium complex binds stronger to DNA than Hoechst dye (2 µM) under the conditions applied (Fig. 4c). The data could not be fitted to obtain accurate Stern–Volmer quenching constants (*K*_sv_). Hoechst-displacement results are compatible with the occurrence of minor groove binding and/or the coordination of the metal complexes to nucleobases. The latter binding mode expels the dye upon shrinkage of the double helix. However, the relatively strong dye displacement induced by **1** and **2**, as compared to those observed with reported minor groove binders [54, 55], rather suggests a nucleobase-coordinating behavior.

#### Circular dichroism (CD)

**Fig. 5:**
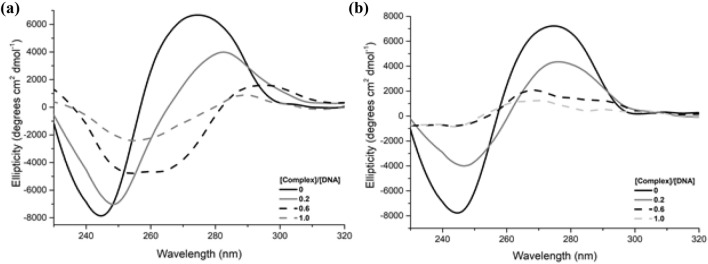
CD spectra of ct-DNA (100 μM) in the absence and presence of **a** [Cu(CPYA)Cl_2_] (**1**) and **b** [Pd(CPYA)Cl_2_] (**2**) at [complex]/[DNA] ratios of 0, 0.2, 0.6 and 1.0

Circular dichroism is a spectroscopic technique that is suitable for the study of structural changes in chiral biomolecules such as amino acids, proteins, RNA and DNA [56]. The CD spectrum of B-DNA exhibits a positive band close to 277 nm that arises from base stacking, and a negative band at 245 nm, which is characteristic of the right-handed helicity [57]. The effect of complexes **1** and **2** on the secondary structure of DNA was investigated at increasing complex:DNA ratios, namely 0.2, 0.6 and 1.0. In the presence of the complexes, the intensity of both the negative and positive bands decreased and a prominent bathochromic shift of both bands occurred (Fig. 5). In the case of **2**, both bands nearly disappeared at complex:DNA ratios of 0.6 onward. These drastic changes on the secondary structure of B-DNA caused by both complexes may arise from strong binding to bases [58].

#### Agarose gel electrophoresis

**Fig. 6:**
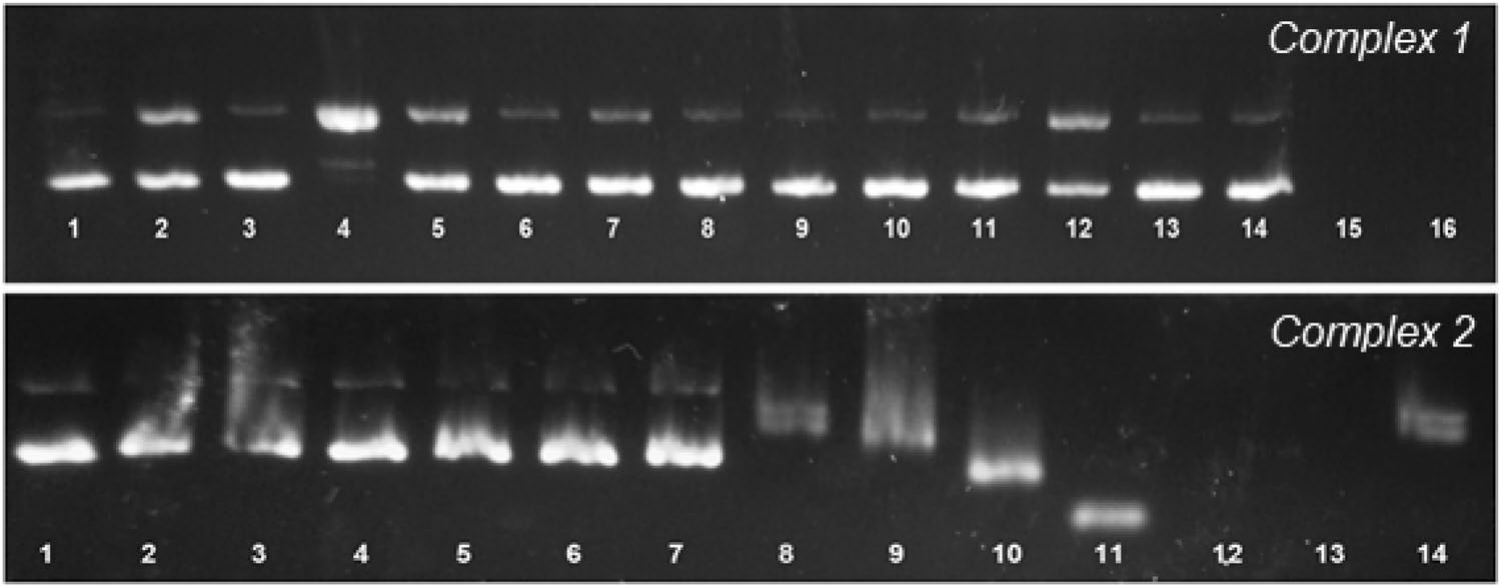
Agarose gel electrophoresis image of 15 µM_bp_ pBR322 plasmid DNA incubated with complexes **1** (1 h, top) and **2** (24 h, bottom) at 37 °C. Top, complex **1**: lane 1: pure plasmid DNA; lane 2: pure plasmid DNA with AA (100 μM); lane 3: CuPhen (5 μM); lane 4: CuPhen with AA (100 μM); lanes 5 and 6: free CPYA (10 and 50 μM); lanes 7 and 8: CPYA with AA; lanes 9–12: [Cu(CPYA)Cl_2_] (**1**) (5, 10, 25 and 50 μM); lanes 13–16: [Cu(CPYA)Cl_2_] (**1**) (5, 10, 25 and 50 μM) with AA. Bottom, complex **2**: lanes 1 and 7: pure plasmid DNA; lanes 8 and 14: cisplatin (10 μM); lanes 2–6: free CPYA (5, 10, 25 and 50 μM); lanes 9–13: [Pd(CPYA)Cl_2_] (**2**) (5, 10, 25, 50 and 100 μM)

The interaction of **1** and **2** with DNA was also visualized by agarose gel electrophoresis. Conformational changes are indeed translated into a different electrophoretic mobility. Oxidative damage to the biomolecule can thus be assessed through the relative amount of cleaved forms that are observed in the gel. Distinct forms of plasmid DNA are typically observed by gel electrophoresis: (a) supercoiled DNA (Form I) that migrates faster on the gel, (b) circular nicked form (Form II) resulting from a one-strand scission, which exhibits a slower migration and (c) linear form (Form III) when both strands are cleaved, which migrates in between forms I and II. Solutions of **1** and **2** of different concentrations were incubated at 37 ºC for 1 h and 24 h, respectively. Two bands are observed for 15 µM_bp_ pure plasmid DNA pBR322, corresponding to the supercoiled form (90%, form I) and circular nicked form (10%, form II), respectively (Fig. 6, lane 1).

The structural changes caused by compound **2** were compared with those of the DNA-binding drug cisplatin (10 µM) (see Fig. 6, bottom). Palladium(II) complex **2** caused a comparable effect to that of cisplatin but at a lower concentration (5 µM). Complex **1** initially caused a decreased mobility of the supercoiled form, which is probably due to an unwinding of the double helix (lane 9). This form seemingly merged with form II, which increased its mobility because of some shrinking upon binding. At higher concentrations of **2**, an increasing shrinkage of both forms resulted in a higher electrophoretic mobility (lanes 10 and 11). At complex-to-DNA base pair ratios above 3, no bands are detected, indicating that the plasmid DNA is fully degraded into undetectable fragments. Such degradation might be due to an extreme tension of the double helix, as a result of strong binding. A similar behavior was reported with a [Pt*L*Cl_2_] complex [26].

**Fig. 7:**
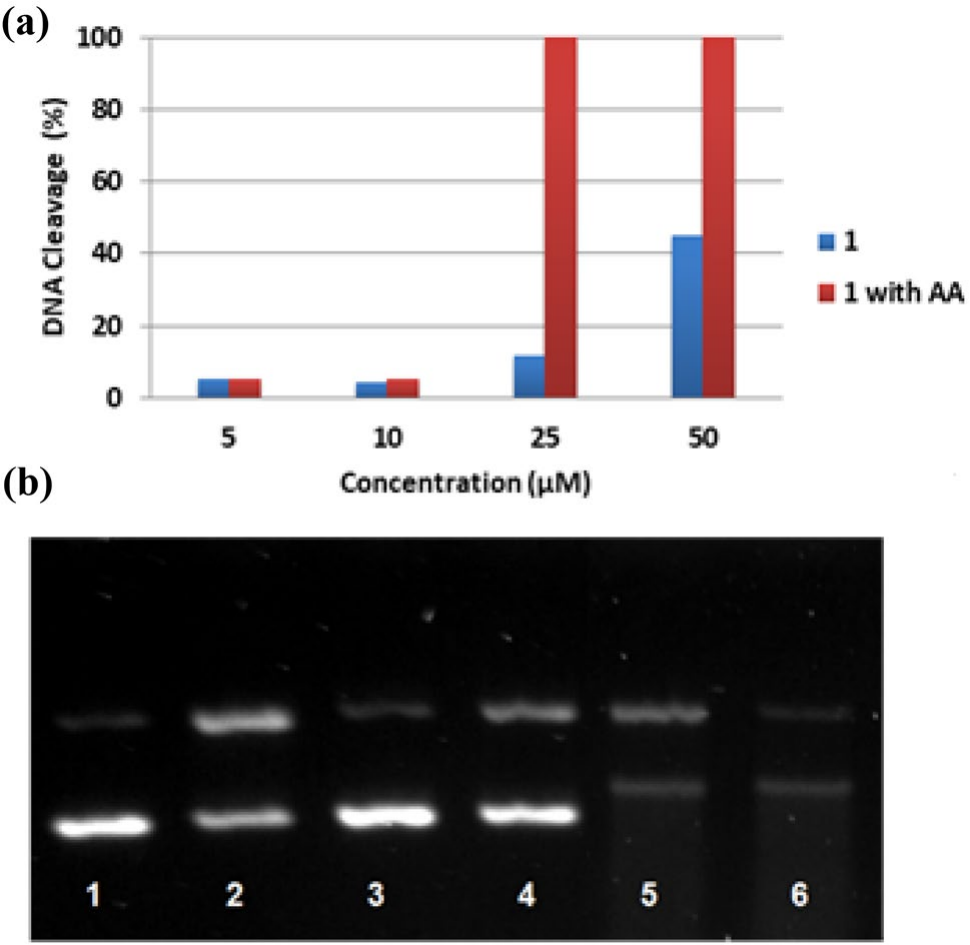
**a** Percentages of DNA cleavage induced by complex [Cu(CPYA)Cl_2_] (**1**) in absence and presence of ascorbic acid (AA, 100 µM). **b** Agarose gel electrophoresis image of 15 µM_bp_ pBR322 plasmid DNA incubated with complex **1** during 1 h at 37 °C showing the effect of different ROS scavengers (20 mM). Lane 1: pure plasmid DNA; lane 2: DNA with AA; lane 3: DNA with AA and NaN_3_; lane 4: DNA with AA and DMSO (20 mM); lane 5: DNA with AA, NaN_3_ and [Cu(CPYA)Cl_2_] (50 μM); lane 6: DNA with AA, DMSO and [Cu(CPYA)Cl_2_] (50 μM)

The effect of copper complex **1** on plasmidic DNA was compared with that of the reference complex [Cu(phen)_2_(H_2_O)](NO_3_)_2_ (CuPhen), also known as Sigman’s reagent, in the presence of a reducing agent, viz., ascorbic acid (AA) (Fig. 6, top) [59]. AA mimics the reducing microenvironment inside the cells and hence enables the Cu(I)/Cu(II) redox cycle, which can produce reactive oxygen species (ROS). AA without metal complex does not cause any DNA damage (lane 2). The metallonuclease CuPhen is not active in the absence of a reducing agent (lane 3). In the presence of AA, CuPhen (5 μM) generates DNA-cleaved forms II and III (lane 4). In absence of AA, **1** does not induce DNA damage at low concentrations (5–25 μM, lanes 9–11). However, a stronger band corresponding to form II is observed at 50 μM (lane 12). A higher degree of cleavage (leading solely to undetectable fragments of the DNA) was observed at complex concentrations of 25 and 50 μM, in the presence of AA (lanes 15 and 16). The percentages of DNA-cleaving activity of **1** were calculated at the concentrations 5, 10, 25 and 50 μM using Eq. (3) (Fig. 7a). The results indicate that the nuclease activity of **1** is remarkable under reducing conditions at complex-to-DNA ratios above 1. This activity is probably due to the generation of reactive oxygen species (ROS) in the proximity of the biomolecule via Fenton-like reaction. ROS can cause the oxidative cleavage of DNA via nucleobase or deoxyribose oxidations [12]. To appraise the nature of the ROS involved in the nuclease activity of **1**, different scavengers, viz., DMSO (for hydroxyl radicals) and sodium azide (for singlet oxygen) were added to various samples containing DNA, **1** and AA, under similar conditions. A significant inhibition of the cleavage activity was observed in the presence of both additives (Fig. 7b) [60]. Complex **1** thus interacts with DNA and cleaves it upon formation of ROS in the presence of a reducing agent, and under aerobic conditions.

Free CPYA did not affect plasmid DNA under the same conditions as those applied for the metal complexes (Fig. 6; lanes 5–8 top and lanes 2–6 bottom).

#### Atomic force microscopy (AFM)

**Fig. 8:**
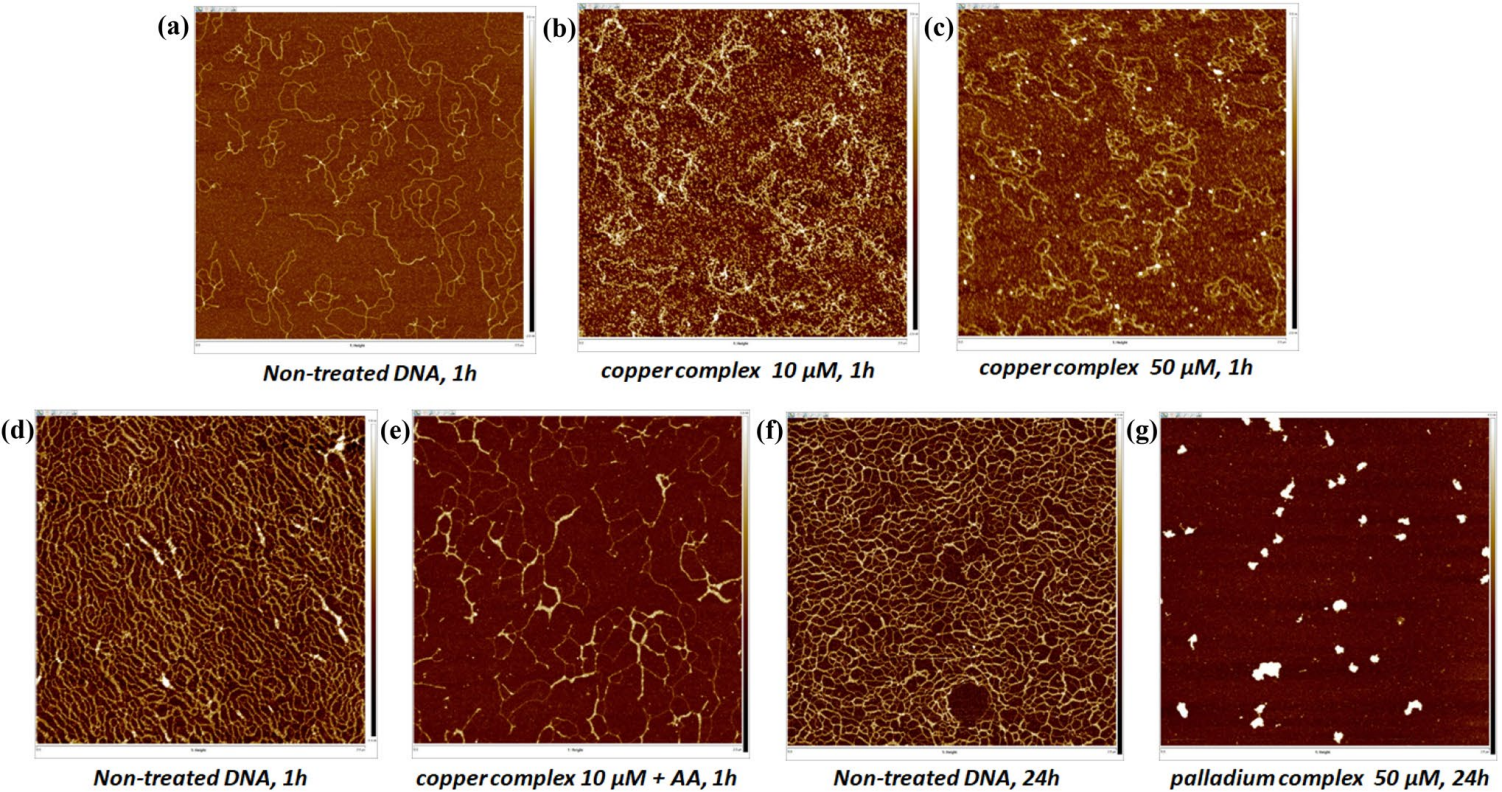
AFM images of plasmid pBR322 DNA (5 and 25 µg mL^–1^) in the absence and presence of various concentrations of complexes **1** and **2**. **a** DNA control; **b** DNA + [Cu(CPYA)Cl_2_] (10 µM); **c** DNA + [Cu(CPYA)Cl_2_] (50 µM); **d** DNA control; **e** DNA + AA (250 µM) + [Cu(CPYA)Cl_2_] (10 µM); **f** DNA control; **g** DNA + [Pd(CPYA)Cl_2_] (50 µM), at 37 ºC in HEPES buffer, pH 7.4 after the respective incubation periods (1 h for **1** and 24 h for **2**)

The effect of the metal complexes on the morphology of pBR322 DNA was investigated by atomic force microscopy (AFM) (Fig. 8), using samples incubated for 1 h with **1** and for 24 h with **2**. The AFM images show that the free plasmid presents well-defined opened forms (Fig. 8a, d and f), which are altered by both complexes. The changes induced by **1** without ascorbic acid are different at 10 and 50 µM (Fig. 8b and c), which reflect its concentration-dependent interaction with the biomolecule (as already observed by UV–Vis spectroscopy; see above). The effect of **1** on the DNA morphology is clearly more pronounced in the presence of ascorbic acid, likely as the result of oxidative damage (Fig. 8e). Regarding Pd(II) complex **2**, the AFM images confirm the spectroscopic data, namely that its interaction with DNA causes drastic changes in the morphology of the biomolecule, ultimately producing double-strand breaks; indeed, small fragments and spherical aggregates can be observed (Fig. 8g) [61]. Similar behaviors have been noticed with other metal-based compounds [50, 62].

### Molecular docking

**Fig. 9:**
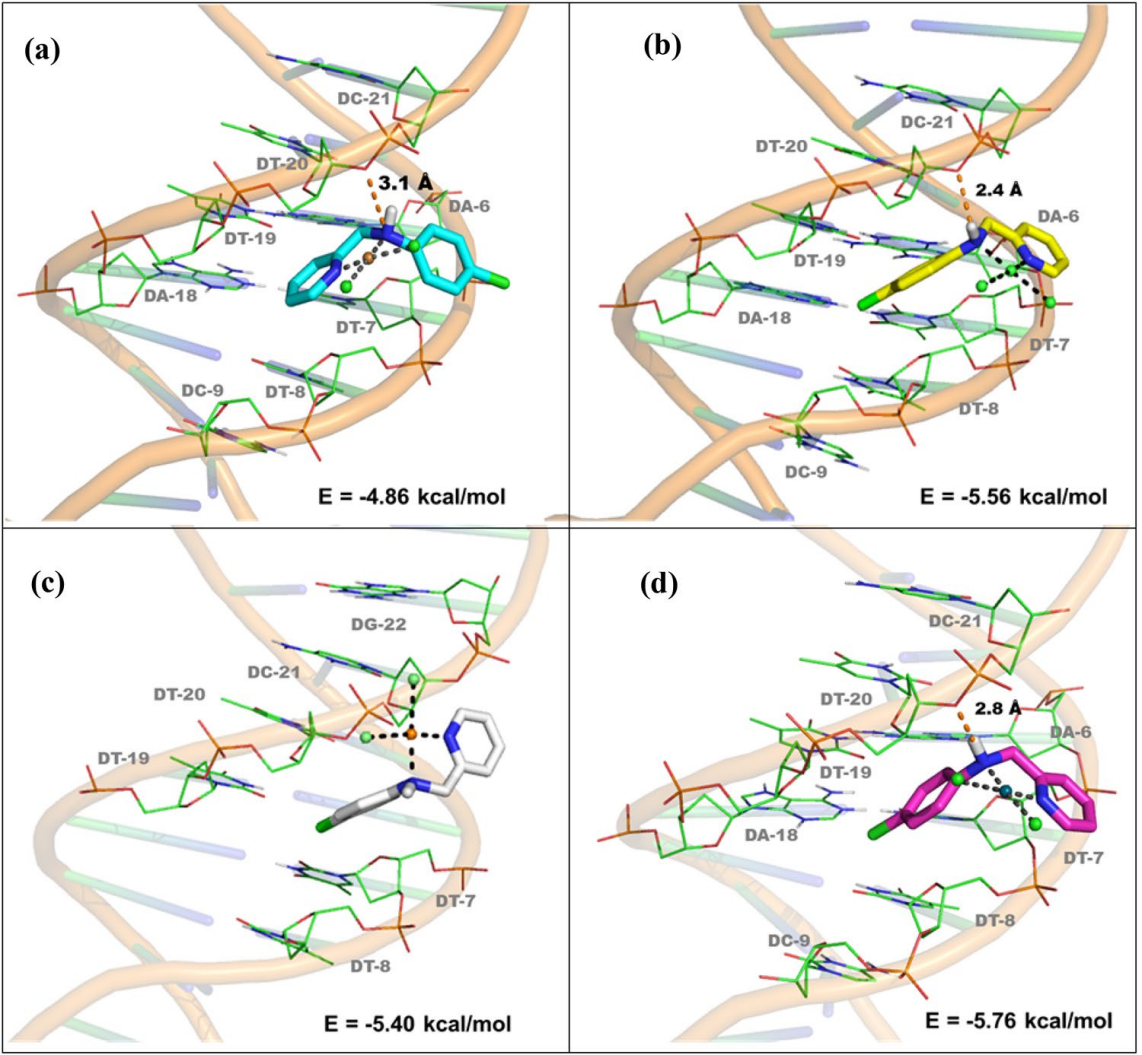
Binding-mode analysis of the two enantiomers of [Cu(CPYA)Cl_2_] (**1**) (top) and [Pd(CPYA)Cl_2_] (**2**) (bottom) with the DNA (PDB: 1BNA). The enantiomers **1R**, **1S**, **2R** and **2S** are shown in cyan, yellow, white and pink, respectively. Hydrogen bonds are colored in orange. Metal–ligand bonds are colored in black

Molecular docking studies were carried out to investigate the potential interactions of racemic [Cu(CPYA)Cl_2_] (**1**) and [Pd(CPYA)Cl_2_] (**2**) in the minor groove of DNA, which would precede binding as indicated by the spectroscopic measurements. Docking studies were conducted with both enantiomers of the complexes (namely, **1R**, **1S**, **2R** and **2S**), since they are expected to interact differently with the chiral double helix. All complexes interact into the minor groove domain via van der Waals interactions, mostly with adenine and thymine nucleobases (Fig. 9). The docking analysis suggests that the different orientations adopted by these compounds are required for their interaction with DNA. For **1R**, the pyridine moiety points directly into the groove, while the chlorophenyl unit points outside this region. The opposite situation is observed for **1S**, for which the chlorophenyl is located into the groove (Fig. 9a and b). The computational studies showed the occurrence of a hydrogen bond between the nitrogen atom of CPYA and an oxygen atom of a deoxythymidine (DT-20), with NH^.…^O distances of 3.1 and 2.4 Å for **1R** and **1S**, respectively. Different features are observed for the palladium complexes. For **2R**, the chlorophenyl moiety is located into the groove and no hydrogen bond is observed (in contrast to **1R**) (Fig. 9c). **2S** presents an orientation that is similar to its copper analog **1S**, including the hydrogen bond, with a NH^.…^O distance of 2.8 Å (Fig. 9d). The binding energies obtained for **1** and **2** are indicative of affinity between these coordination compounds and the DNA double helix. The results point toward a higher affinity for the palladium(II) complex (− 5.40 and − 5.76 kcal/mol) when compared to that of copper(II) (− 4.86 and − 5.56 kcal/mol), in accordance with the spectroscopic, electrophoretic and AFM data.

**Fig. 10:**
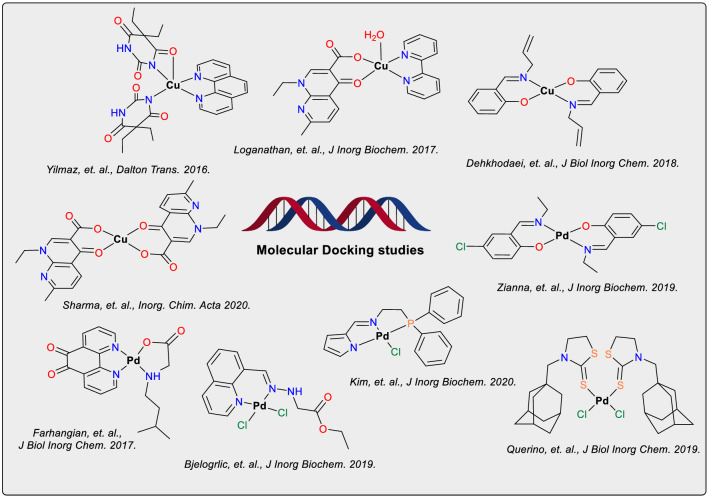
Structures of different copper- and palladium-based complexes for which molecular docking studies of their interaction with DNA were reported

Several copper and palladium-based compounds have been reported with a similar behavior (Fig. 10). The molecular docking results with DNA show that these coordination compounds also interact in the minor groove [20, 60, 63, 65–69]. Although the van der Waals contacts appear to be pivotal for the interaction with this groove, hydrogen bonding with nucleobases seems to be important for the stabilization into the domain [69].

Since experimental data suggested the presence of aquo species, docking studies were also performed for both enantiomers of the aquated complexes [Cu(CPYA)(H_2_O)_2_]^2+^ (**1a**) and [Pd(CPYA)(H_2_O)_2_]^2+^ (**2a**) (namely **1aR**, **1aS**, **2aR** and **2aS**) (Fig. S13). All docked poses interact into the groove and different orientations are adopted by the aquo species, as observed for the chloride complexes (**1** and **2**). Although the interactions mostly occur via van der Waals contacts, hydrogen bonds are observed as well between the water ligands and the phosphate groups of the sugar–phosphate backbone. A similar behavior using aquo complexes (instead of chloride complexes) was observed by Querino et al. with different platinum(II) and palladium(II) complexes (Fig. 10) [69]. In these studies, besides the van der Waals contacts, the authors also observed the interaction of the palladium(II) complex with phosphate groups, further stabilizing its binding into the groove.

For the copper(II) complexes, hydrogen bonds with distances of 1.9, 2.0 and 2.1 Å, respectively, were detected for **1aR** (Fig. S13a). In contrast, no hydrogen bonds were observed for **1aS** (Fig. S13b). Regarding the palladium(II) complexes, hydrogen bonds with distances of 2.4 and 2.1 Å, were found for **2aR** and **2aS**, respectively (Figs. S13c and S13d). As observed with the initial chloride species (i.e., **1** and **2**), the higher binding energies are achieved with the palladium compounds (− 11.12 and − 11.42 kcal/mol). It should be highlighted that all the energy values obtained are comparable to those previously reported in the literature for related copper(II) and palladium(II) complexes, namely in the range of −5.50 to −10.12 kcal/mol [20, 64, 67, 68].

## Conclusions

Two *cis*-chlorido square-planar complexes have been prepared from the *N*,*N*-donor ligand 4-chloro-*N*-(pyridin-2-ylmethyl)aniline (CPYA) and two different metal ions, namely copper(II) and palladium(II). The interaction of [Cu(CPYA)Cl_2_] (**1**) and [Pd(CPYA)Cl_2_] (**2**) with DNA was subsequently investigated. The data achieved illustrate the crucial role played by the metal center; although the coordination environment is comparable for both complexes, their binding to the biomolecule is clearly different. For instance, the mode of interaction of the copper complex with DNA is concentration dependent, while it is not the case for that of palladium. The palladium complex **2** strongly binds to the biomolecule (in contrast to complex **1**), inducing important structural changes. Thus, shrinking is observed with **2** at low concentrations and aggregation occurs at higher concentrations, eventually leading to the cleavage of the double strand into small fragments. Copper complex **1** predominantly interacts electrostatically with the DNA minor groove at high complex-to-DNA ratios, while binding appears to take place at lower complex-to-DNA ratios. Moreover, the redox-active metal generates ROS in the presence of a reducing agent, giving rise to the oxidative cleavage of DNA (at high complex concentrations). Molecular docking studies suggest that van der Waals forces drive the initial interaction/recognition of both complexes in the minor groove of the double helix, but with slight differences depending on the metal center, again illustrating their key role.

## Supplementary Information

Below is the link to the electronic supplementary material.

Supplementary file1 (PDF 1128 KB)

## References

[1] Rosenberg B, van Camp L, Krigas T (1965). Nature.

[2] Rosenberg B, van Camp L, Grimley EB, Thomson AJ (1967). J Biol Chem.

[3] Kelland L (2007). Nature Rev Cancer.

[4] Johnstone TC, Suntharalingam K, Lippard SJ (2016). Chem Rev.

[5] Wheate NJ, Walker S, Craig GE, Oun R (2010). Dalton Trans.

[6] Santini C, Pellei M, Gandin V, Porchia M, Tisato F, Marzano C (2014). Chem Rev.

[7] Alam MN, Huq F (2016). Coord Chem Rev.

[8] Meier-Menches SM, Gerner C, Berger W, Hartinger CG, Keppler BK (2018). Chem Soc Rev.

[9] Mari C, Pierroz V, Ferrari S, Gasser G (2015). Chem Sci.

[10] Renfrew AK, O'Neill ES, Hambley TW, New E (2018). Coord Chem Rev.

[11] Bonnet S (2018). Dalton Trans.

[12] Brissos RF, Caubet A, Gamez P (2015). Eur J Inorg Chem.

[13] Erxleben A (2018). Coord Chem Rev.

[14] Zoroddu MA, Aaseth J, Crisponi G, Medici S, Peana M, Nurchi VM (2019). J Inorg Biochem.

[15] Fanelli M, Formica M, Fusi V, Giorgi L, Micheloni M, Paoli P (2016). Coord Chem Rev.

[16] Matilla A, Tercero JM, Niclos-Gutierrez J, Dung N-H, Voissat B, Perez JM, Alonso C, Martin-Ramos JD (1994). J Inorg Biochem.

[17] Omondi RO, Bellam R, Ojwach SO, Jaganyi D, Fatokun AA (2020). J Inorg Biochem.

[18] Oliveira CG, Romero-Canelón I, Silva MM, Coverdale JPC, Maia PIS, Batista AA, Castelli S, Desideri A, Sadler PJ, Deflon VM (2019). Dalton Trans.

[19] Quiroga AG, Pérez JM, Montero EI, Masaguer JR, Alonso C, Navarro-Ranninger C (1998). J Inorg Biochem.

[20] Bjelogrlić SK, Todorović TR, Kojić M, Senćanski M, Nikolić M, Višnjevac A, Araškovc J, Miljković M, Muller CD, Filipović NR (2019). J Inorg Biochem.

[21] Rau T, Alsfasser R, Zahl A, van Eldik R (1998). Inorg Chem.

[22] Hannon MJ (2007). Pure Appl Chem.

[23] Barrera-Guzman VA, Rodriguez-Hernandez EO, Ortiz-Pastrana N, Dominguez-Gonzalez R, Caballero AB, Gamez P, Barba-Behrens N (2018). J Biol Inorg Chem.

[24] Grau J, Caubet A, Roubeau O, Montpeyó D, Lorenzo J, Gamez P (2020). ChemBioChem.

[25] Barra CV, Rocha FV, Gautier A, Morel L, Quilles MB, Carlos IZ, Treu-Filho O, Frem RCG, Mauro AE, Netto AVG (2013). Polyhedron.

[26] Grau J, Renau C, Caballero AB, Caubet A, Pockaj M, Lorenzo J, Gamez P (2018). Dalton Trans.

[27] Park S, Lee JK, Lee H, Nayab S, Shin JW (2019). Appl Organomet Chem.

[28] Ahn SH, Lee H (2016). Bull Korean Chem Soc.

[29] Fernandes CM, Mello MVP, Santos NE, Souza AMT, Lanznaster M, Ponzio EA (2020). Mater Corros.

[30] Brauer G (1965). Handbook of preparative inorganic chemistry.

[31] Bruker (2017). APEX3v2017.3-0.

[32] Bruker (2017). SAINT.v8.38A.

[33] Sheldrick GM (1996) SADABS, Program for Area Detector Adsorption Correction. Institute for Inorganic Chemistry, University of Göttingen, Germany

[34] Sheldrick GM (2008). Acta Crystallogr Sect A.

[35] Sheldrick GM (2015). Acta Cryst.

[36] Dolomanov OV, Bourhis LJ, Gildea RJ, Howard JAK, Puschmann H (2009). J Appl Cryst.

[37] Macrae CF, Edgington PR, McCabe P, Pidcock E, Shields GP, Taylor R, Towler M, van de Streek J (2006). J Appl Cryst.

[38] Catalan KJ, Jackson S, Zubkowski JD (1995). Polyhedron.

[39] Morris GM, Huey R, Lindstrom W, Sanner MF, Belew RK, Goodsell DS, Olson AJ (2009). J Comput Chem.

[40] Türkkan E, Sayin U, Erbilen N, Pehlivanoglu S, Erdogan G, Tasdemir HU, Saf AO, Guler L, Akgemci EG (2017). J Organomet Chem.

[41] Posada NBM, Guimarães NA, Padilha DS, Resende JALC, Faria RB, Lanznaster M, Amado RS, Scarpellini M (2018). Polyhedron.

[42] Mandal M, Oppelt K, List M, Teasdale I, Chakraborty D, Monkowius U (2016). J Biol Inorg Chem.

[43] Choroba K, Machura B, Szlapa-Kula A, Malecki JG, Raposo L, Roma-Rodrigues C, Cordeiro S, Baptista PV, Fernandes AR (2021). Eur J Med Chem.

[44] Małecki JG, Maron A (2011). Trans Met Chem.

[45] Mandal S, Naskar B, Modak R, Sikdar Y, Chatterjee S, Biswas S, Mondal TK, Modak D, Goswami S (2015). J Mol Struct.

[46] Yang L, Powell DR, Houser RP (2007). Dalton Trans.

[47] Fakih S, Tung WC, Eierhoff D, Mock C, Krebs B (2005). Z Anorg Allg Chem.

[48] Kellett A, Molphy Z, Slator C, McKee V, Farrell NP (2019). Chem Soc Rev.

[49] Pages BJ, Ang DL, Wright EP, Aldrich-Wright JR (2015). Dalton Trans.

[50] Censi V, Caballero AB, Perez-Hernandez M, Soto-Cerrato V, Korrodi-Gregorio L, Perez-Tomas R, Dell'Anna MM, Mastrorilli P, Gamez P (2019). J Inorg Biochem.

[51] Křikavová R, Vančo J, Trávníček Z, Hutyra J, Dvořák Z (2016). J Inorg Biochem.

[52] Parsekar SU, Fernandes J, Banerjee A, Chouhan OP, Biswas S, Singh S, Mishra DP, Kumar M (2018). J Biol Inorg Chem.

[53] Yang P, Zhang D, Wang Z, Liu H, Shi Q, Xie X (2019). Dalton Trans.

[54] Jalali F, Dorraji PS (2017). Arab J Chem.

[55] Kabeer H, Hanif S, Arsalan A, Asmat S, Younus H, Shakir M (2020). ACS Omega.

[56] Nordén B, Rodger A, Dafforn T (2010). Linear dichorism and circular dichroism: a textbook on polarized light spectroscopy.

[57] Ivanov VI, Minchenkova LE, Schyolkina AK, Poletayev AI (1973). Biopolymers.

[58] Fu X-B, Liu D-D, Lin Y, Hu W, Mao Z-W, Le X-Y (2014). Dalton Trans.

[59] McGivern TJP, Afsharpour S, Marmion CJ (2018). Inorg Chim Acta.

[60] Loganathana R, Ganeshpandiana M, Bhuvanesh NSP, Palaniandavara M, Muruganantham A, Ghosh SK, Riyasdeen A, Akbarsha MA (2017). J Inorg Biochem.

[61] Mounir M, Lorenzo J, Ferrer M, Prieto MJ, Rossell O, Avilès FX, Moreno V (2007). J Inorg Biochem.

[62] Grau J, Brissos RF, Salinas-Uber J, Caballero AB, Caubet A, Roubeau O, Korrodi-Gregório L, Pérez-Tomás R, Gamez P (2015). Dalton Trans.

[63] Yilmaz VT, Icsel C, Suyunova F, Aygun M, Aztopal N, Ulukaya E (2016). Dalton Trans.

[64] Dehkhodaei M, Sahihi M, Rudbari HA, Momenbeik F (2018). J Biol Inorg Chem.

[65] Sharma M, Ganeshpandian M, Sanjeev A, Tamilarasan A, Mattaparthi VSK, Islam NS, Palaniandavar M (2020). Inorg Chim Acta.

[66] Zianna A, Geromichalos GD, Pekou A, Hatzidimitriou AG, Coutouli-Argyropoulou E, Lalia-Kantouri M, Pantazaki AA, Psomas G (2019). J Inorg Biochem.

[67] Farhangian H, Moghadam ME, Divsalar A, Rahiminezhad A (2017). J Biol Inorg Chem.

[68] Kim Y, Lee J, Son Y-H, Choi S-U, Alam M, Park S (2020). J Inorg Biochem.

[69] Querino ALA, Silva JT, Silva JT, Alvarenga GM, Silveira CH, Magalhães MTQ, Chaves OA, Iglesias BA, Diniz R, Silva H (2019). J Biol Inorg Chem.

