# Supplementary material for: DNA-interacting properties of two analogous square-planar cis-chlorido complexes: copper versus palladium
Source: J Biol Inorg Chem. 2021 Aug 28;26(6):727–40. doi: 10.1007/s00775-021-01888-2 (PMC8437883; doi:10.1007/s00775-021-01888-2)
Supplement: Supplementary file 1 — Supplementary file1 (PDF 1128 KB) [file 775_2021_1888_MOESM1_ESM.pdf]

# Supplementary Information

# DNA-interacting properties of two analogous square-planar *cis*-chlorido complexes: copper *versus* palladium


Marcos V. Palmeira-Mello[a,b,c], Ana B. Caballero[b],* Aida Lopez Espinar[b], Guilherme P. Guedes[a], Amparo Caubet[b], Alessandra M. Teles de Souza[c], Mauricio Lanznaster[a] and Patrick Gamez[b,d,e]

[a] *Instituto de Química, Universidade Federal Fluminense, Outeiro S. João Batista S/N, 24020−141, Niterói RJ, Brazil.*

[b] *nanoBIC, Departament de Química Inorgànica I Orgànica, Secció Química Inorgànica, Universitat de Barcelona, Martí i Franquès 1−11, 08028 Barcelona, Spain.*

[c] *Laboratório de Modelagem Molecular & QSAR (ModMolQSAR), Faculdade de Farmácia, Universidade Federal do Rio de Janeiro, Rio de Janeiro, RJ, Brazil.*

[d] *Institute of Nanoscience and Nanotechnology (IN2UB), Universitat de Barcelona, 08028 Barcelona, Spain.*

[e] *Catalan Institution for Research and Advanced Studies (ICREA), Passeig Lluís Companys 23, 08010 Barcelona, Spain.*

*Corresponding author: Ana B. Caballero, ana.caballero@ub.edu

# Index of Figures and Tables

**Fig. S1** Infrared spectrum (diamond/ZnSe ATR) of **1**.

**Fig. S2** Infrared spectrum (diamond/ZnSe ATR) of **2**.

**Fig. S3** $^{1}H$ NMR spectrum of **2** in DMSO-$d_6$ and zoom of the region 9.0–4.4 ppm (inset).

**Fig. S4** ESI-MS spectrum of **1**.

**Fig. S5** ESI-MS spectrum of **2**.

**Fig. S6** UV-Visible spectra of **1** in DMSO at different concentrations.

**Fig. S7** UV-Visible spectra of **2** in DMSO at different concentrations.

**Fig. S8** UV-Visible monitoring of **1** (50 μM) in cacodylate–NaCl buffer containing 1% DMSO during 1 h at room temperature.

**Fig. S9** UV-Visible monitoring of **2** (50 μM) in cacodylate–NaCl buffer containing 1% DMSO during 24 h at room temperature.

**Fig. S10** $^{1}H$ NMR spectra of **2** in DMSO and DMSO/$D_2O$ after 0, 1 and 24 h at 37 ºC.

**Fig. S11** Fluorescence emission spectra of the DNA–EB complex in the absence and presence of increasing amounts of complex **1**, [EB] = 75 μM, [DNA] =15 $\mu M_{bp}$, [Complex] = 0–50 μM, $\lambda_{ex}$ = 514 nm.

**Fig. S12** Fluorescence emission spectra of the DNA–EB complex in the absence and presence of increasing amounts of complex **2**, [EB] = 75 μM, [DNA] =15 $\mu M_{bp}$, [Complex] = 0–50 μM, $\lambda_{ex}$ = 514 nm.

**Fig. S13** Binding-mode analysis of the two enantiomers of $[Cu(CPYA)(H_2O)_2]^{2+}$ (**1a**) (top) and $[Pd(CPYA)(H_2O)_2]^{2+}$ (**2a**) (bottom) with the DNA (PDB: 1BNA). The enantiomers **1a*R***, **1a*S***, **2a*R*** and **2a*S*** are shown in cyan, yellow, white and pink, respectively. Hydrogen bonds are colored in orange. Metal-ligand bonds are colored in gray.

**Table S1** Summary of crystal data, collection and refinement of complexes $[Cu(CPYA)Cl_2]$ (**1**) and $[Pd(CPYA)Cl_2]$ (**2**).

**Table S2** Selected bond lengths (Å) and angles (°) for complexes $[Cu(CPYA)Cl_2]$ (**1**) and $[Pd(CPYA)Cl_2]$ (**2**).

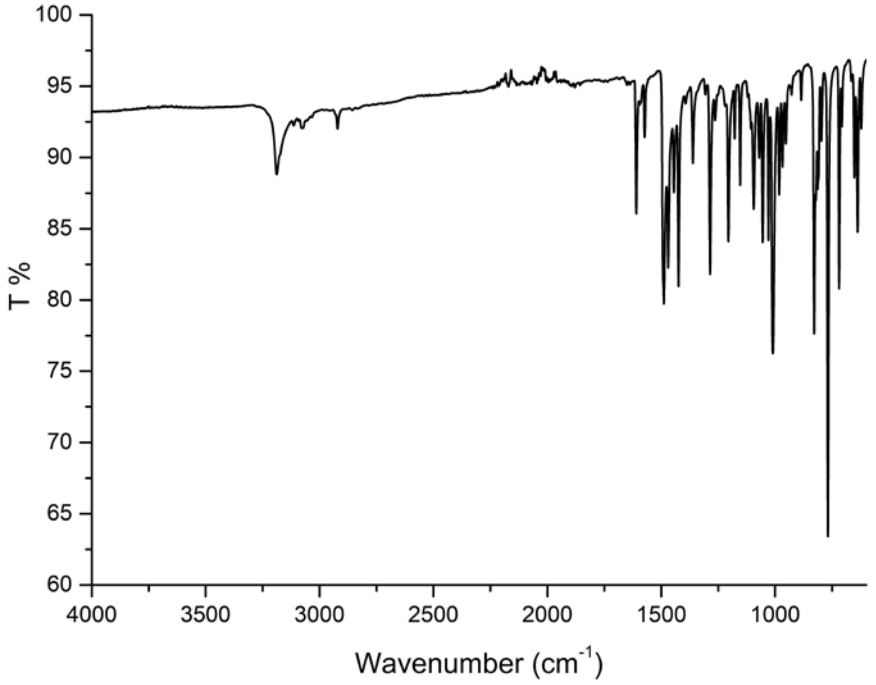


**Fig. S1** Infrared spectrum (diamond/ZnSe ATR) of **1**.

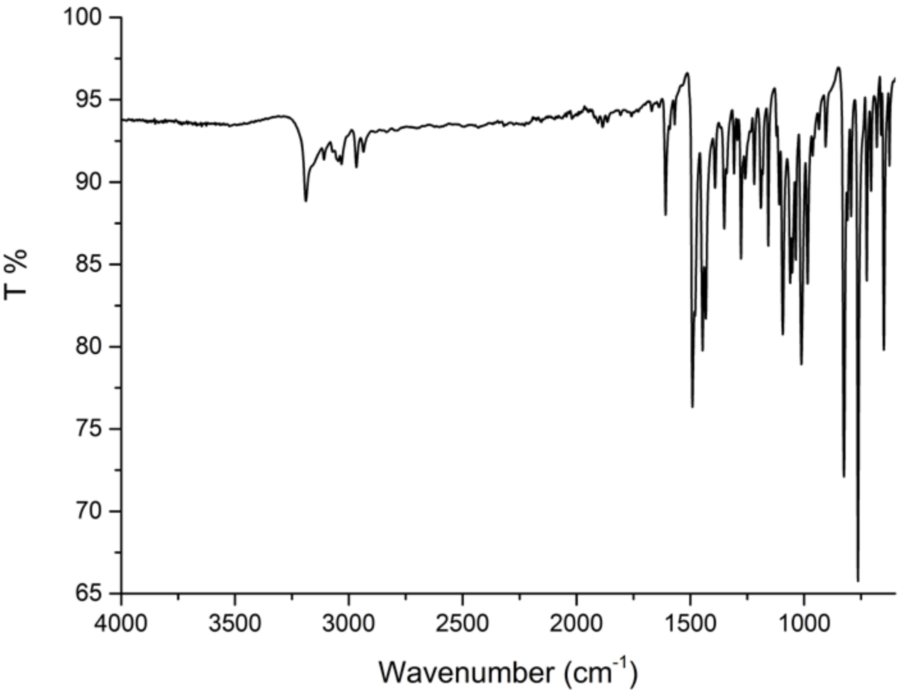


**Fig. S2** Infrared spectrum (diamond/ZnSe ATR) of **2**.

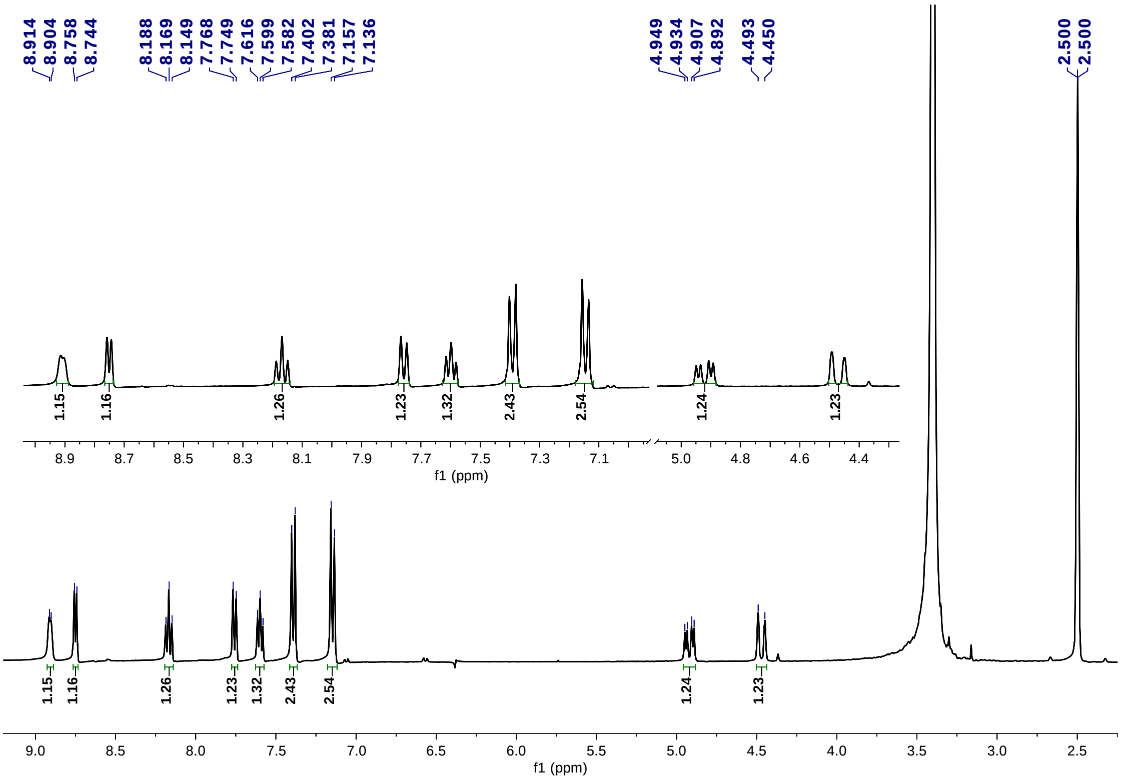


**Fig. S3** $^1H$ NMR spectrum of **2** in DMSO-d$_6$ and zoom of the region 9.0–4.4 ppm (inset).

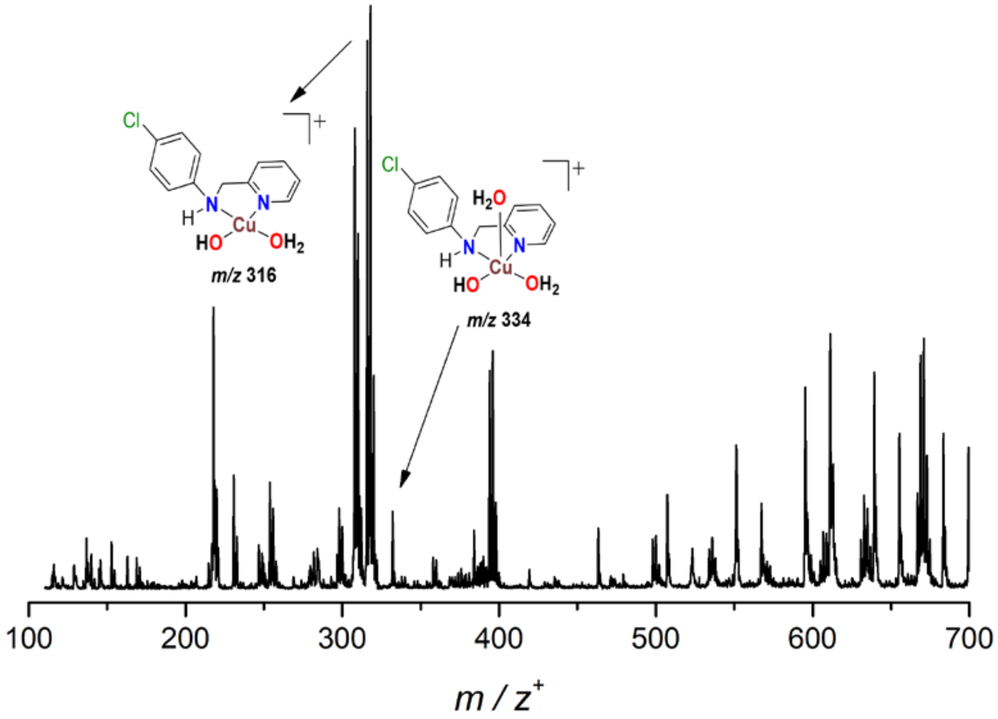


**Fig. S4** ESI-MS spectrum of **1**.

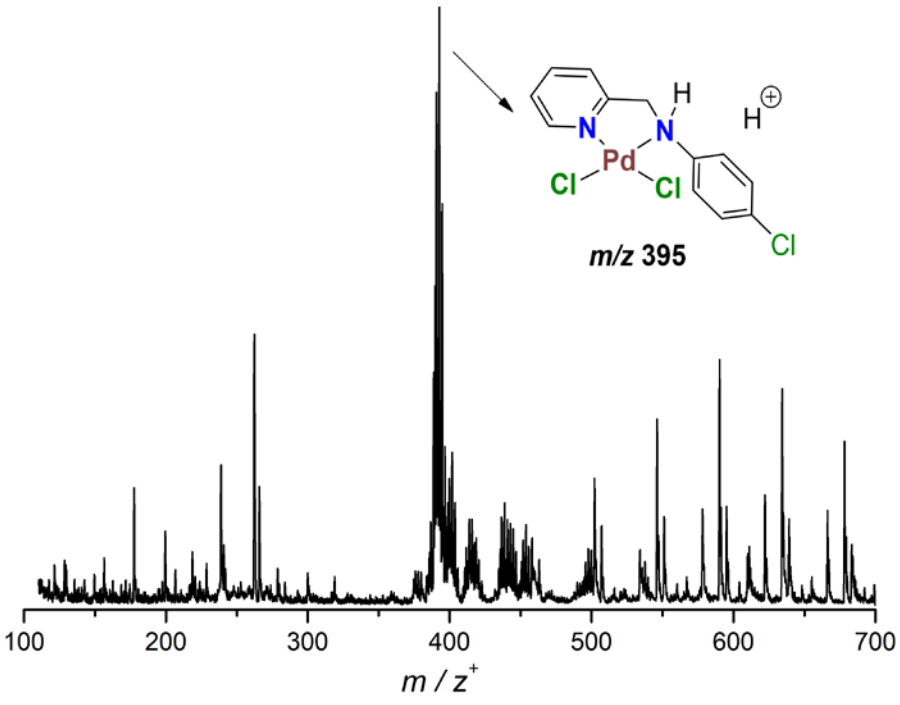


**Fig. S5** ESI-MS spectrogram of **2**.

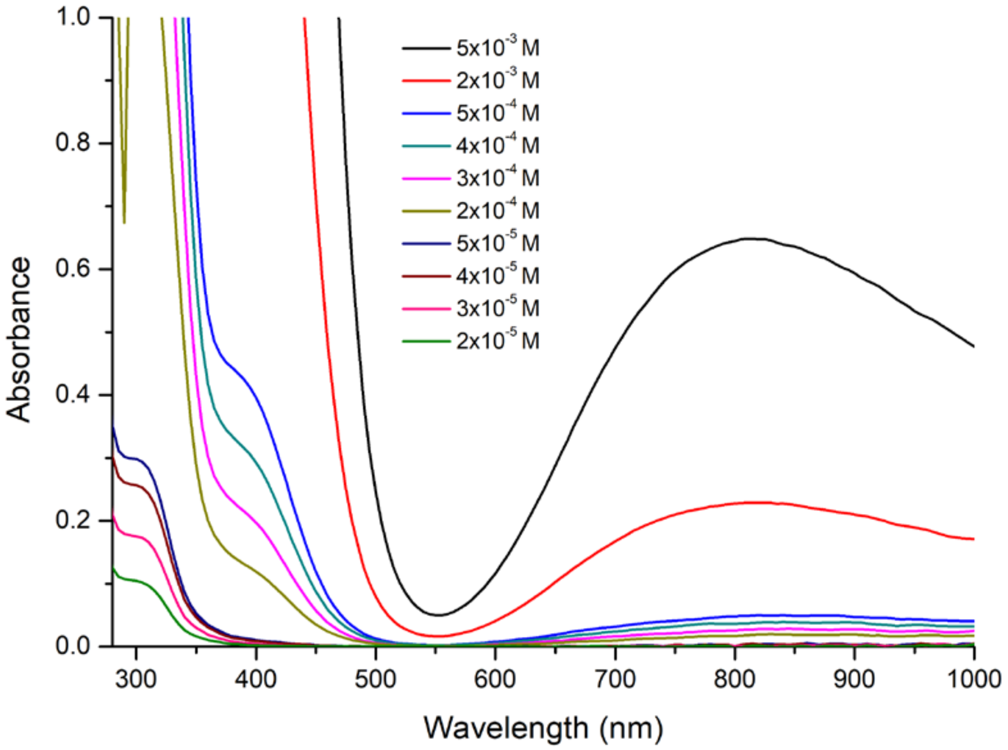


**Fig. S6** UV-Visible spectra of **1** in DMSO at different concentrations.

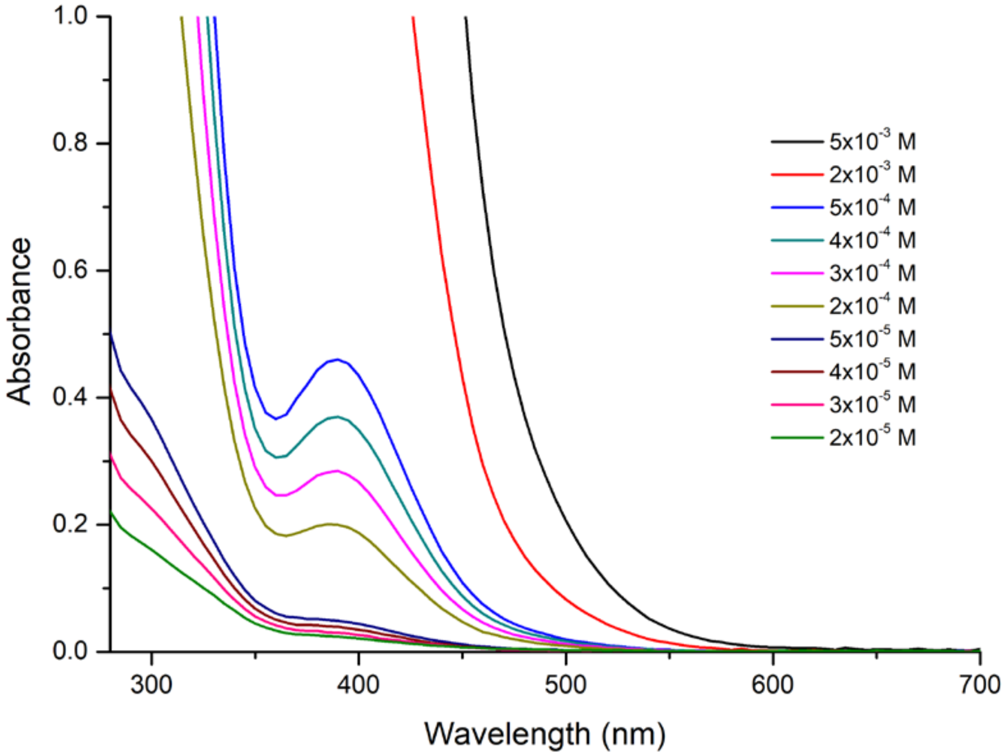


**Fig. S7** UV-Visible spectra of **2** in DMSO at different concentrations.

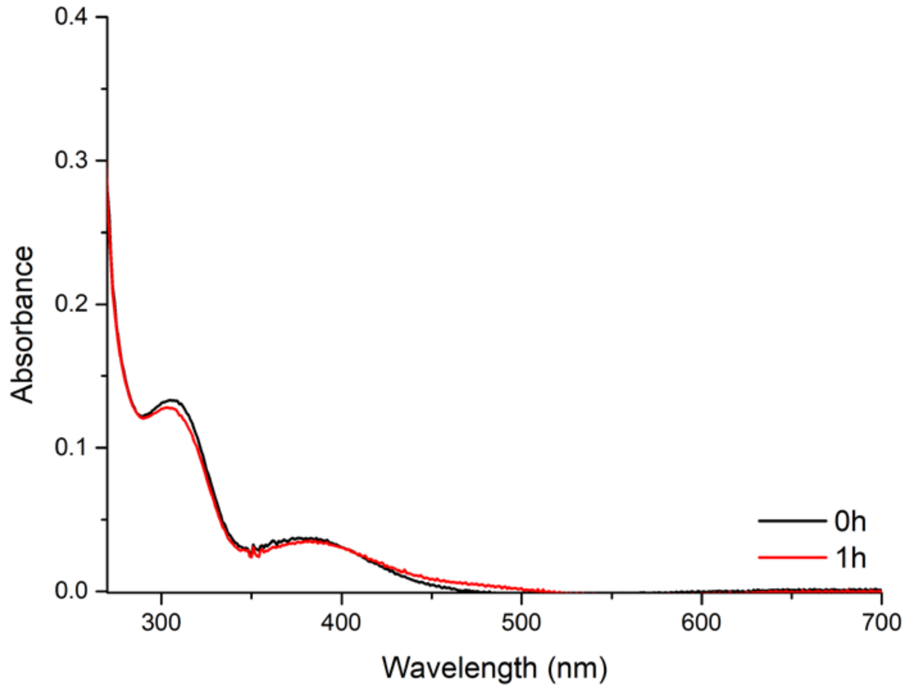


**Fig. S8** UV-Visible monitoring of **1** (50 μM) in cacodylate–NaCl buffer containing 1% DMSO during 1 h at room temperature.

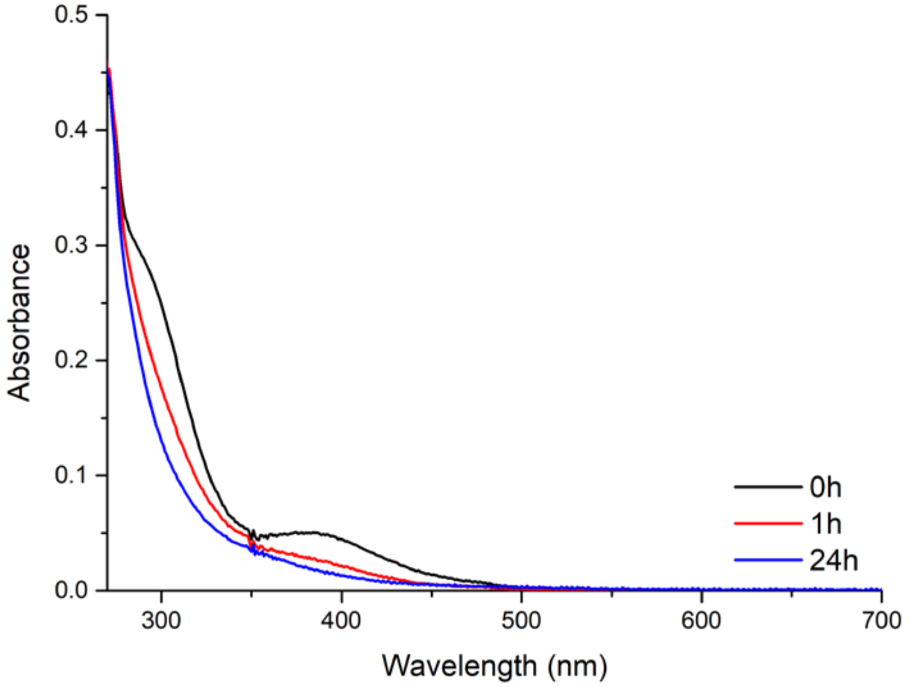


**Fig. S9** UV-Visible monitoring of **2** (50 μM) in cacodylate–NaCl buffer containing 1% DMSO during 24 h at room temperature.

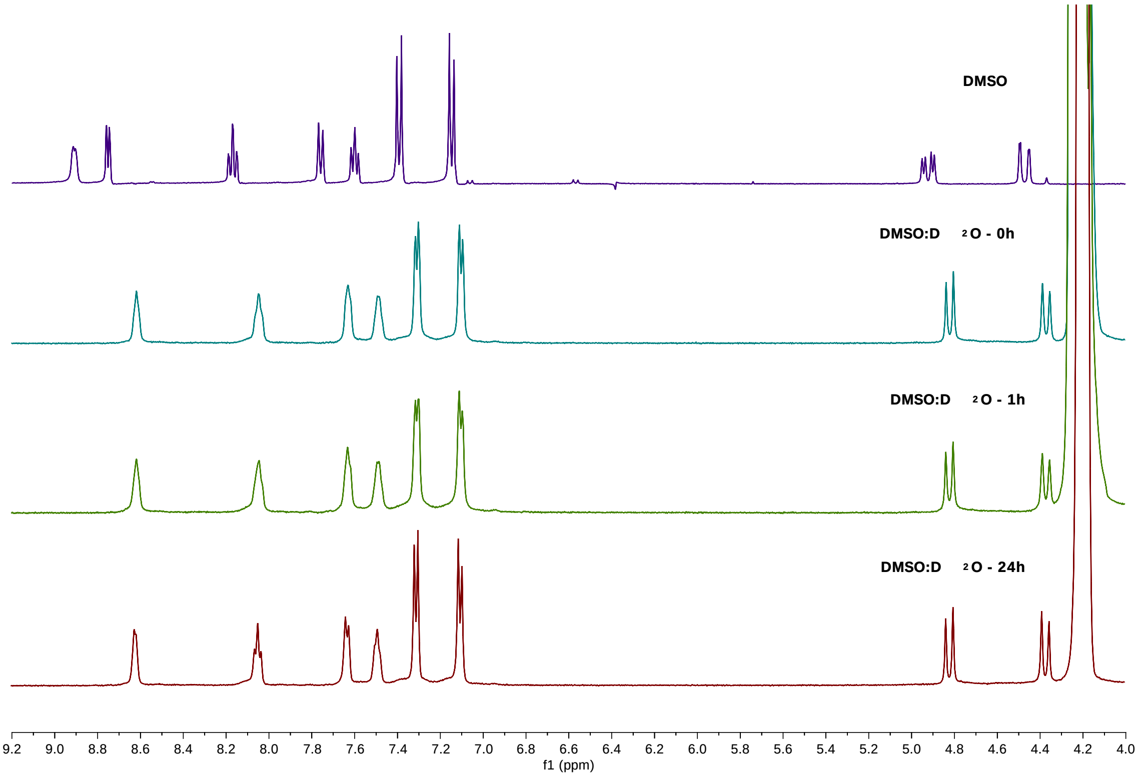


**Fig. S10** $^{1}$H NMR spectra of **2** in DMSO and DMSO/$D_2O$ after 0, 1 and 24 h at 37 ºC.

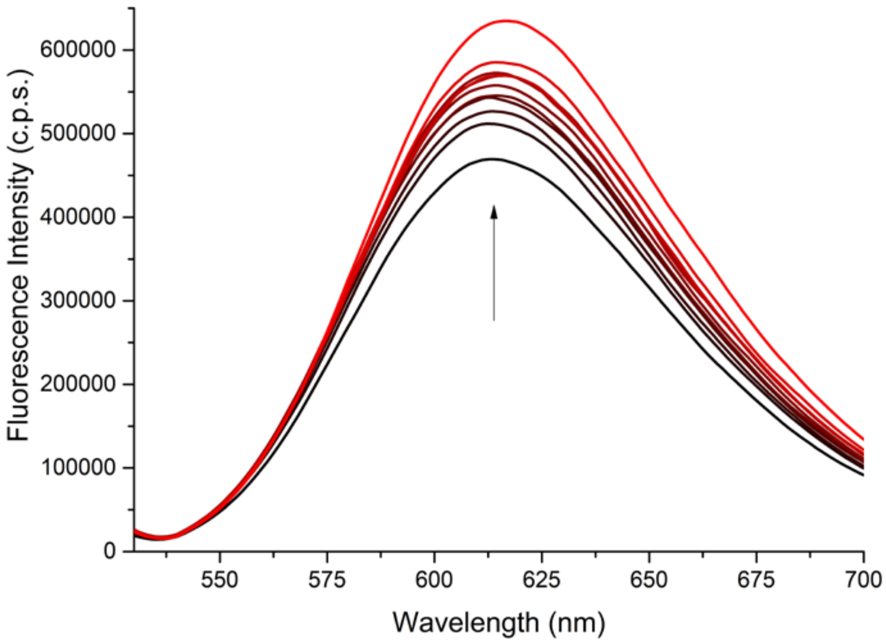


**Fig. S11** Fluorescence emission spectra of the DNA–EB complex in the absence and presence of increasing amounts of complex **1**, [EB] = 75 µM, [DNA] =15 µM, [Complex] = 0–50 µM, $\lambda_{ex}$ = 514 nm.

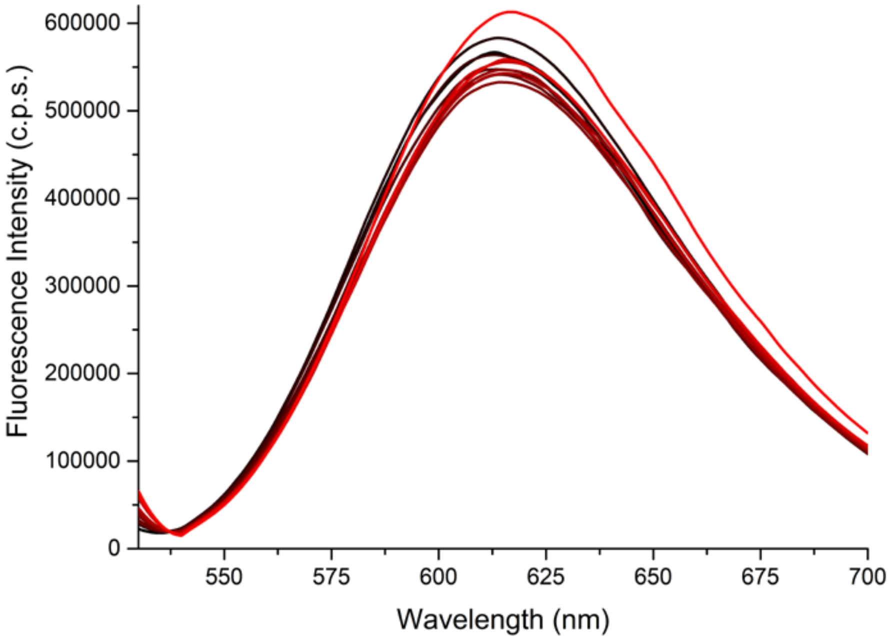


**Fig. S12** Fluorescence emission spectra of the DNA–EB complex in the absence and presence of increasing amounts of complex **2**, [EB] = 75 µM, [DNA] =15 µM, [Complex] = 0–50 µM, $\lambda_{ex}$ = 514 nm.

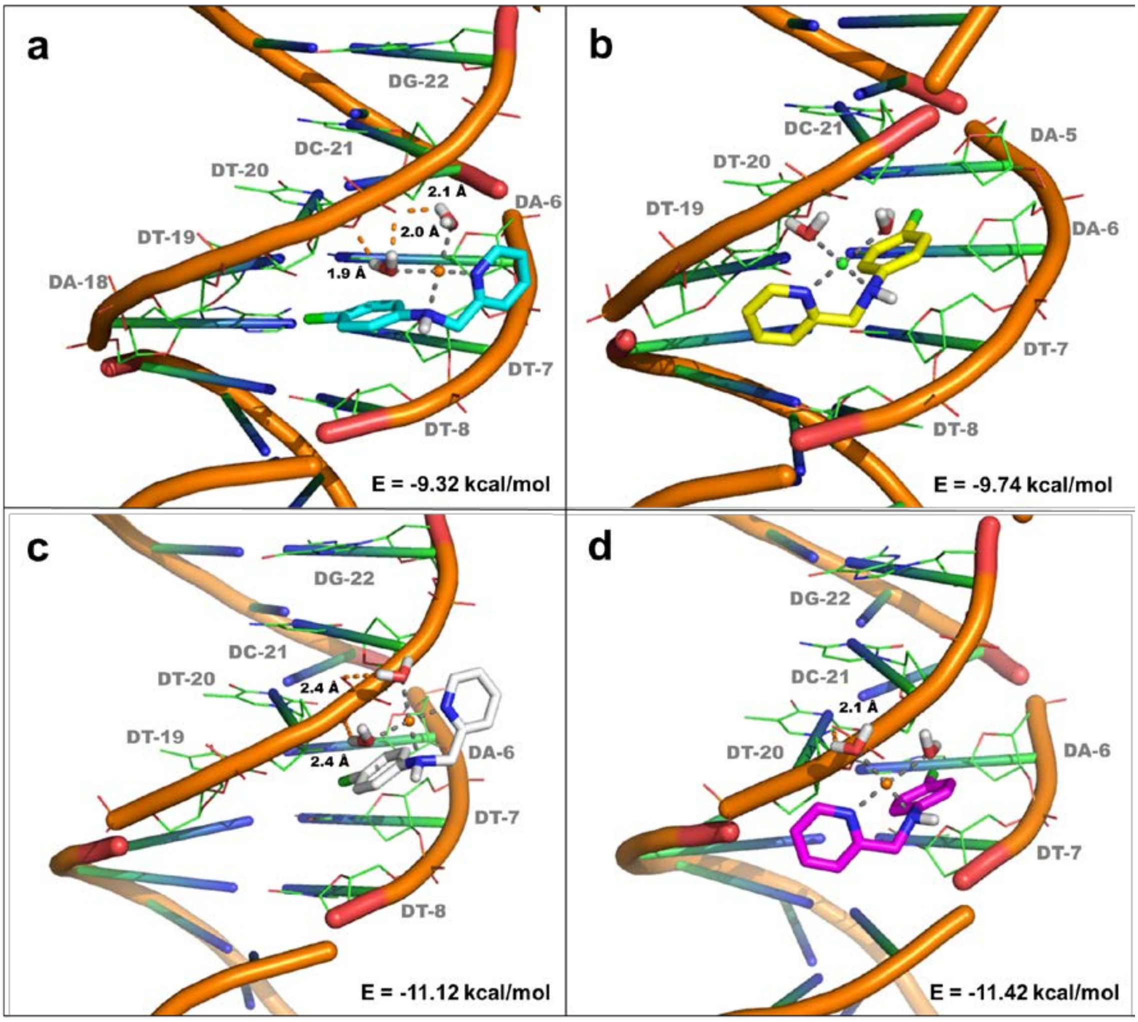


**Fig. S13** Binding-mode analysis of the two enantiomers of $[Cu(CPYA)(H_2O)_2]^{2+}$ (**1a**) (top) and $[Pd(CPYA)(H_2O)_2]^{2+}$ (**2a**) (bottom) with the DNA (PDB: 1BNA). The enantiomers **1a*R***, **1a*S***, **2a*R*** and **2a*S*** are shown in cyan, yellow, white and pink, respectively. Hydrogen bonds are colored in orange. Metal-ligand bonds are colored in gray.

**Table S1** Summary of crystal data, collection and refinement of complexes [Cu(CPYA)$Cl_2$] (**1**) and [Pd(CPYA)$Cl_2$] (**2**).

| | *Complex **1*** | *Complex **2*** |
|---|---|---|
| Empirical formula | $C_{12}H_{11}Cl_3CuN_2$ | $C_{12}H_{11}Cl_3N_2Pd\cdot CH_3CN$ |
| Formula weight (g.mol$^{-1}$) | 353.12 | 437.03 |
| Crystal system | Monoclinic | Monoclinic |
| Space group | $P2_1/c$ | $P2_1/c$ |
| Temperature (K) | 298 | 298 |
| Wavelength | Mo Kα | Mo Kα |
| Unit cell dimensions (Å,º) | a = 12.2234 (13)<br>b = 14.8800 (14)<br>c = 7.7366 (6)<br>α = 90°<br>β = 105.775 (4)°<br>γ = 90° | a = 10.744 (4)<br>b = 9.369 (3)<br>c = 16.417 (6)<br>α = 90°<br>β = 92.169 (13)°<br>γ = 90° |
| Volume (Å$^3$) | 1354.2 (2) | 1651.4 (10) |
| Z | 4 | 4 |
| $D_x$ (Mg/m$^3$) | 1.732 | 1.758 |
| Absorption coefficient (mm$^{-1}$) | 2.19 | 1.60 |
| F(000) | 708 | 864 |
| Index ranges | h = −14 → 14<br>k = −17 → 17<br>l = −9 → 9 | h = −12 → 12<br>k = −11 → 11<br>l = −19 → 19 |
| $R_{int}$ | 0.036 | 0.073 |
| Reflections collected | 38635 | 26680 |
| Independent reflections | 2380 | 2887 |
| Reflections observed [I>2σ(I)] | 2102 | 2525 |
| ϴ range for data collection | 3.1 to 25.0º | 2.5 to 25.0º |
| R [$F^2>2\sigma F^2$] | 0.025 | 0.054 |
| wR ($F^2$) | 0.059 | 0.146 |
| S | 1.14 | 1.15 |
| Parameters | 168 | 191 |
| Δρ min (e Å$^{-3}$) | −0.30 | −1.37 |
| Δρ max (e Å$^{-3}$) | 0.44 | 1.92 |

**Table S2** Selected bond lengths (Å) and angles (°) for complexes [Cu(CPYA)$Cl_2$] (**1**) and [Pd(CPYA)$Cl_2$] (**2**).

| *Atom labels* | **1** (M = Cu) | **2** (M = Pd) |
|---|---|---|
| M—N1 | 2.087(2) | 2.081(5) |
| M—N2 | 1.999(2) | 2.040(5) |
| M—Cl1 | 2.2524(7) | 2.2979(18) |
| M—Cl2 | 2.2645(7) | 2.3142(17) |
| | | |
| N1—M—N2 | 82.40(8) | 82.64(19) |
| N1—M—Cl1 | 90.19(6) | 90.43(14) |
| N1—M—Cl2 | 177.28(6) | 175.73(14) |
| N2—M—Cl1 | 168.95(6) | 172.64(14) |
| N2—M—Cl2 | 95.03(6) | 93.99(14) |
| Cl1—M—Cl2 | 92.23(3) | 93.04(6) |
